# Selective protein aggregation confines and inhibits endotoxins in wounds: Linking host defense to amyloid formation

**DOI:** 10.1016/j.isci.2023.107951

**Published:** 2023-09-19

**Authors:** Jitka Petrlova, Erik Hartman, Ganna Petruk, Jeremy Chun Hwee Lim, Sunil Shankar Adav, Sven Kjellström, Manoj Puthia, Artur Schmidtchen

**Affiliations:** 1 Division of Dermatology and Venereology, Department of Clinical Sciences, Lund University, 22184 Lund, Sweden; 2 School of Materials Science and Engineering, Nanyang Technological University, Singapore, Singapore; 3 Department of Clinical Sciences, BioMS, Lund University, Lund, Sweden; 4 Dermatology, Skane University Hospital, 22185 Lund, Sweden

**Keywords:** Immunology, Bacteriology, cell biology

## Abstract

Bacterial lipopolysaccharide (LPS) induces rapid protein aggregation in human wound fluid. We aimed to characterize these LPS-induced aggregates and their functional implications using a combination of mass spectrometry analyses, biochemical assays, biological imaging, cell experiments, and animal models. The wound-fluid aggregates encompass diverse protein classes, including sequences from coagulation factors, annexins, histones, antimicrobial proteins/peptides, and apolipoproteins. We identified proteins and peptides with a high aggregation propensity and verified selected components through Western blot analysis. Thioflavin T and Amytracker staining revealed amyloid-like aggregates formed after exposure to LPS *in vitro* in human wound fluid and *in vivo* in porcine wound models. Using NF-κB-reporter mice and IVIS bioimaging, we demonstrate that such wound-fluid LPS aggregates induce a significant reduction in local inflammation compared with LPS in plasma. The results show that protein/peptide aggregation is a mechanism for confining LPS and reducing inflammation, further emphasizing the connection between host defense and amyloidogenesis.

## Highlights

• LPS triggers protein aggregation in wound fluid, confining and reducing inflammation
• Subsets of protein classes form LPS-induced aggregates in wound fluid
• Aggregates reduce inflammation via NF-κB inhibition *in vitro* and *in vivo*
• Study uncovers link between host defense, inflammation, and amyloidogenesis

## Graphical abstract

**Figure:**
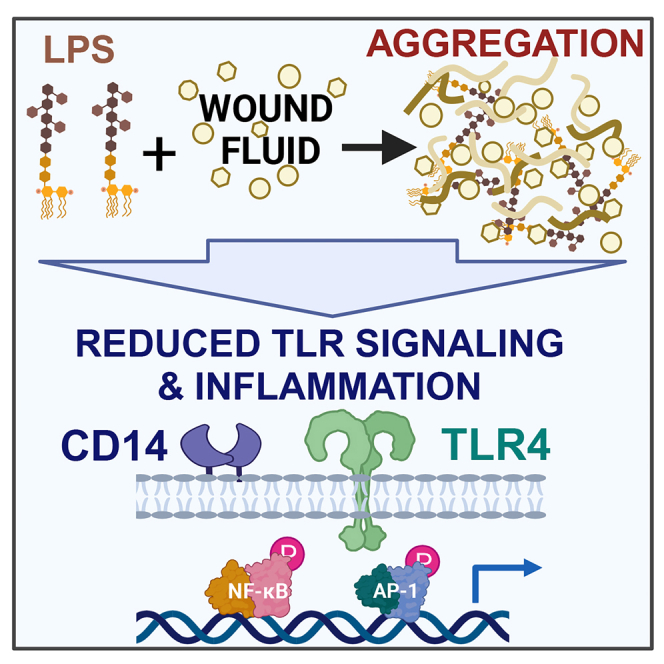

## Introduction

Skin wounds pose a potential threat for bacterial invasion and sepsis, and a multitude of host defense systems have consequently evolved. These include coagulation, initial hemostasis, and the subsequent action of multiple proteins and peptides of our innate immune system.^1^^,^^2^ Examples are neutrophil-derived α-defensins and the cathelicidin LL-37,^2^^,^^3^^,^^4^ histones,^3^^,^^5^^,^^6^ lysozyme,^3^ and proteolytic products of plasma proteins, such as fibrinogen,^7^ complement factor C3,^8^ and thrombin.^9^^,^^10^

Lipopolysaccharide (LPS) sensing by Toll-like receptor 4 (TLR4) controls early responses to infection. However, an excessive LPS response is deleterious, causing localized inflammation such as that found in infected wounds, as well as severe systemic responses such as those seen in sepsis.^11^ Therefore, clearance and control of endotoxins are critical for a robust antibacterial response while maintaining control of inflammatory responses.

We have previously demonstrated that addition of LPS or bacteria to human acute wound fluids (AWFs) leads to precipitation of protein aggregates containing C-terminal thrombin fragments of about 11 kDa, which mediate LPS aggregation and scavenging.^12^^,^^13^ These observations led us to hypothesize that aggregation in wounds constitutes an early sensing and clearance mechanism to control and scavenge excessive LPS levels during wounding and injury. However, it was unknown whether other proteins and peptides apart from the identified thrombin fragments could participate in LPS-scavenging in human AWFs.

Thus, we sought to define the LPS interactome in AWF and the functional consequences of LPS aggregation. Using mass spectrometry analysis, we show that such aggregates not only contain thrombin sequences, but also other coagulation factors and sequences from protein families, including annexins, histones, antimicrobial proteins/peptides, and apolipoproteins. Proteins and peptides with a high propensity for aggregation were identified, demonstrating a subclass of LPS-interacting molecules in AWFs. Selected aggregating components were verified biochemically by Western blot analysis. Staining by thioflavin T and the Amytracker probe demonstrates the presence of amyloid-like aggregates formed after exposure to LPS *in vitro* in human AWF, as well as *in vivo* in porcine wounds. Using NF-κB-reporter mice and *In Vivo* Imaging System (IVIS) bioimaging, we show that such LPS aggregates in AWF induce a significant reduction in local inflammation when compared with LPS administrated with plasma.

## Results

### General experimental outline

**Figure 1:**
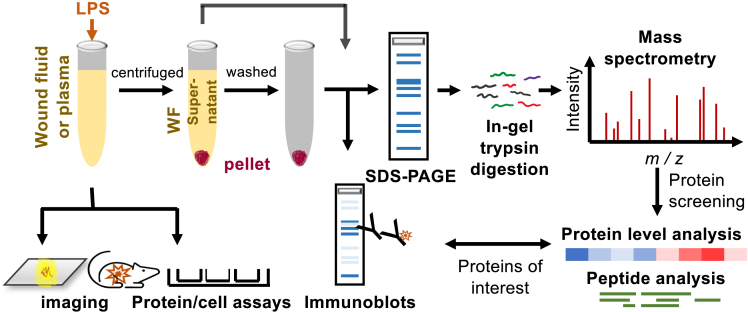
Experimental design Sample preparation of acute wound fluids (AWFs), plasma, and pig wound fluid for imaging, analysis, various assays and mass spectrometry.

The main outline is shown in Figure 1. Briefly, to examine whether LPS induces aggregation and amyloid formation in AWFs, we analyzed the supernatant and pellet by biochemical, imaging, and mass spectrometry methodologies. Quantitative analyses were done to measure aggregating proteins, and qualitative studies were done using mass spectrometry analysis of proteomes, Western blot of selected proteins, and analyses of the peptidomes generated. Finally, functional analyses were done using *in vitro* and *in vivo* analyses of LPS-induced NF-κB activation and the effects of LPS confinement by aggregation.

### LPS induces aggregation in WFs *in vitro* and in wounds *in vivo*

**Figure 2:**
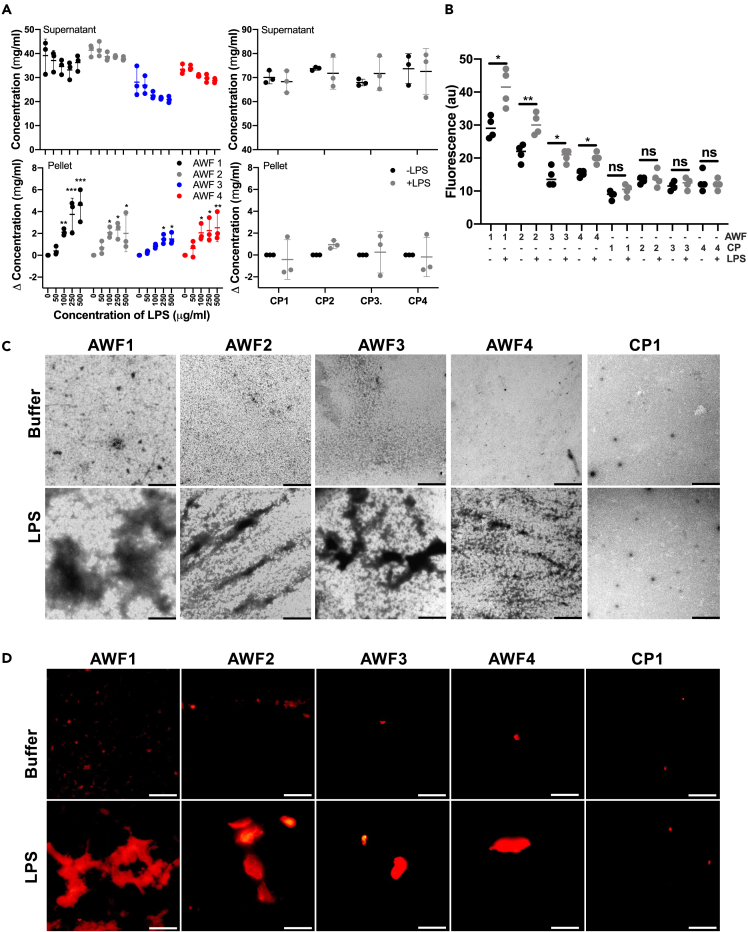
Protein aggregation in AWFs challenged by LPS (A) Analysis of protein content after addition of LPS to acute wound fluids (AWFs). Various doses of LPS were added to AWFs and protein content determined by BCA assay. A significant increase of protein content was detected in the pellets of the AWFs. No increase was observed in the citrated plasma (CP) samples. The graphs are presented as means ± SD from at least three independent experiments. ∗p ≤ 0.05; ∗∗p ≤ 0.01; and ∗∗∗p ≤ 0.001. p values were determined relative untreated (control) using one-way ANOVA followed by Dunnett’s multiple comparisons test. (B) ThT aggregation assay showed a significant increase of aggregates in all AWFs after addition of 100 μg/mL of LPS. No such effects were seen in the CP samples. Statistical analysis was performed using one-way ANOVA with Dunnett’s multiple comparison tests from four independent experiments (n = 4). ∗ = p ≤ 0.05 and ∗∗ = p ≤ 0.01. (C) TEM – negative stain showed amorphous aggregates in all AWFs exposed to LPS (100 μg/mL). The images represent an example from three independent experiments. The scale bar is 5 μm. (D) Fluorescence microscopy using Amytracker 680 staining shows increase of stained aggregates in AWFs after addition of LPS (100 μg/mL). The images represent an example from three independent experiments. The scale bar is 5 μm.

Four AWFs were incubated with *E. coli* LPS, and the protein content in the resulting pellets and supernatants was analyzed. Overall, we detected a significant increase in protein amounts in pellets of the AWFs after incubation with 100–500 μg/mL of LPS (Figure 2A, panels on the left). Notably, an increase in the protein amount in the pellet corresponded to a decrease in the supernatants. No such LPS-induced changes were observed in the four citrate plasma (CP) samples analyzed (Figure 2A, panels on the right). Furthermore, we observed a significant decrease in the concentration of LPS in the supernatants of centrifuged LPS-treated AWFs using the limulus amebocyte lysate (LAL) assay. Conversely, there was an increase in LPS content detected in the pellets of centrifuged LPS-treated AWFs (Figure S1A). Moreover, using the fluorescent dye thioflavin T1 (ThT), we detected a significant increase in fluorescence after addition of LPS to the AWFs, but no difference was observed for the plasma samples (Figure 2B). ThT specifically binds to β-sheet structures in aggregating proteins and amyloids, so the results demonstrated that LPS caused a significant increase in amyloid-like aggregates in all four AWFs analyzed (Figure 2B). To investigate whether AWFs could aggregate with other LPS-types, we used LPS from *Pseudomonas aeruginosa*. Using the ThT assay, we detected a similar increase in fluorescence when the AWFs were exposed to *P. aeruginosa* LPS (Figure S1B, panel on the left). Lipid A, the lipid core of both LPS-types, did not yield any increase in ThT fluorescence (Figure S1B, panel on the right), indicating the importance of the polysaccharide part of LPS for formation of amyloid aggregates.

Next, we employed transmission electron microscopy (TEM) with negative stain to visualize the formed protein aggregates, and the results showed that *E. coli* LPS induced formation of aggregates in all AWFs, which were not seen in the CP sample (Figure 2C). A similar result was obtained using *P. aeruginosa* LPS (Figure S1C). Amytracker 680 is a fluorescent tracer for high-quality visualization of protein aggregation and amyloids. Using the fluorescent probe, we detected a significant increase in fluorescence signal in all AWFs subjected to 100 μg/mL of *E. coli* LPS, which again contrasted with the results obtained using CP (Figures 2D and S2A). Buffer and LPS alone yielded a low fluorescence signal (Figure S2B). FITC-labeled LPS was used to study its presence within the aggregates. In a similar experiment to the one mentioned previously, fluorescence microscopy imaging revealed the presence of FITC label within the aggregates, which were also stained by Amytracker 680. This staining combination indicated that FITC-LPS is indeed being scavenged within the amyloid aggregates (Figure S3).

**Figure 3:**
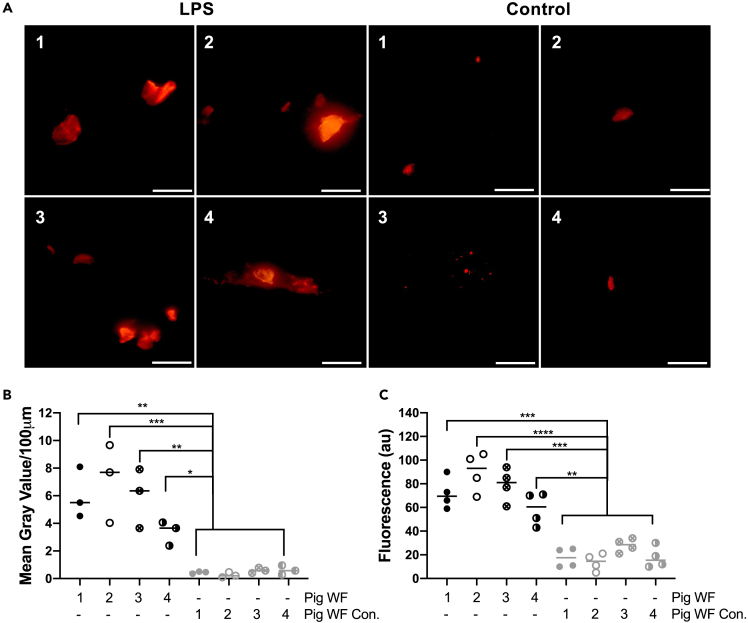
Protein aggregation in porcine wounds *in vivo* (A) Fluorescence microscopy analysis of samples stained with Amytracker 680 stain shows an increase of aggregates in all AWFs derived from wounds exposed to LPS (100 μg/mL). The images represent an example from three independent experiments. The scale bar is 5 μm. (B) Image analyses of Amytracker 680 signal. (C) ThT aggregation assay confirmed a significant increase of aggregates in all AWFs from wounds treated with 100 μg/mL of LPS. No aggregates were detected in the control WFs. Statistical analysis was performed using one-way ANOVA with Dunnett’s multiple comparison tests from four independent experiments (n = 4). ∗ = p ≤ 0.05, ∗∗ = p ≤ 0.01, ∗∗∗ = p ≤ 0.001 and ∗∗∗∗ = p ≤ 0.0001.

We next explored whether AWF aggregates can also be formed *in vivo*. For this purpose, we employed a partial thickness wound model in Göttingen minipigs. In a reductionist approach, we used topical LPS application, thus avoiding possible confounders induced by bacterial infection per se. Using Amytracker, we detected a significant increase in aggregates in WFs from LPS-treated wounds in comparison to untreated controls (Figures 3A and 3B). Likewise, using ThT staining, a higher number of amyloid aggregates were detected in the LPS-treated wounds (Figure 3C). Taken together, the results demonstrate that LPS induces protein aggregation *in vitro* in human AWFs and *in vivo* in porcine wounds and that the aggregates have amyloid-like properties.

### Mass spectrometry analysis of LPS-induced AWF aggregates

We next performed a proteomic analysis of the components found in the aggregates with an overall aim of determining whether certain components are prone to aggregation with LPS. For this, protein pellets and supernatants from AWFs aggregated with LPS were separated on SDS-PAGE and prepared for LC-MS/MS analysis. Peptides from four biological replicates and their technical duplicates were separated and analyzed to determine the specificity of LPS-triggered protein aggregation and enrichment in the pellet.

**Figure 4:**
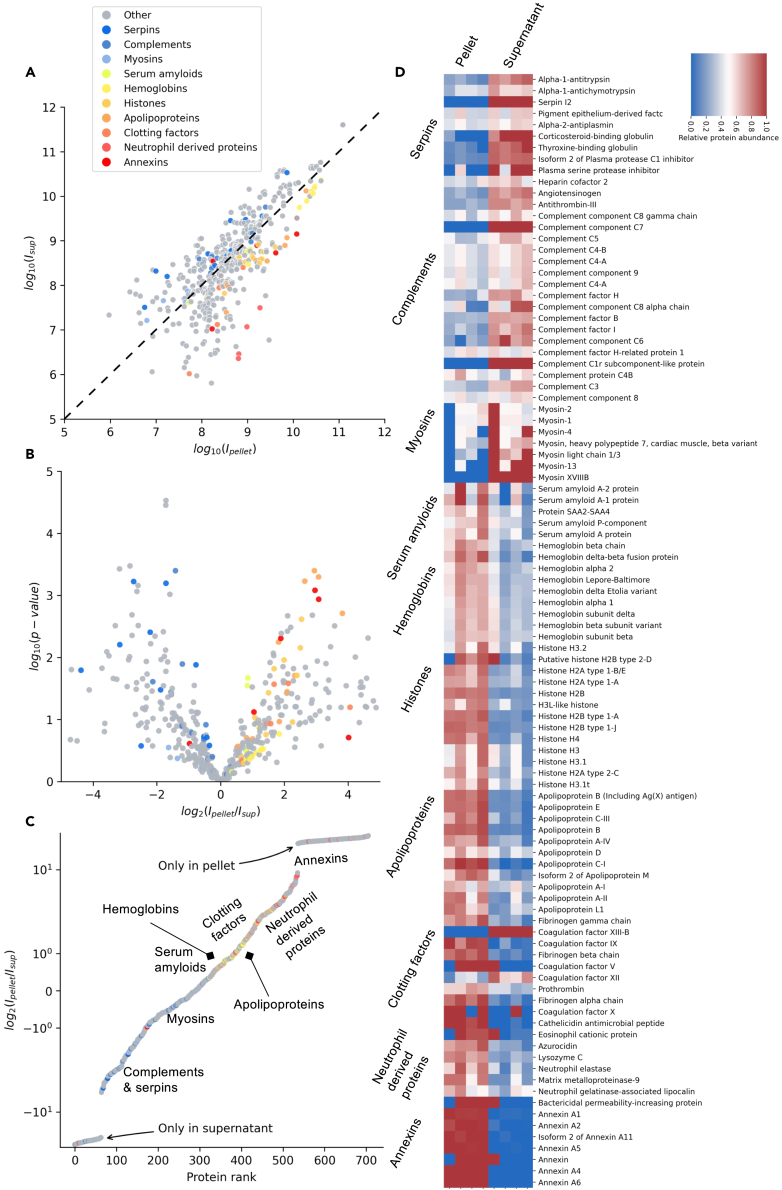
Mass spectrometry proteomic analysis of the LPS aggregatome (A) Protein MS intensities derived from pellets and supernatants of all four AWFs treated with LPS. Each specific protein is represented as a dot in the graph. Proteins which are not at all present in one of the groups are excluded. The dashed diagonal represents equal abundance in both samples. Proteins are colored if they are a part of a relevant protein group. (B) A volcano plot of the proteins in the supernatant and pellet. The volcano plot shows the log-transformed p value over the log-transformed fold change of average protein intensities. Proteins are colored if they are a part of a relevant protein group. (C) The log-transformed fold change between pellet and supernatant over the protein rank. Here, the proteins solely present in the pellet and supernatant respectively are included, as contrary to A and B where they are outside the bounds of the axes’ limits. Proteins are colored if they are a part of a relevant protein group. (D) Heatmap of selected proteins based on the relative protein abundance in the pellet and the supernatant in AWFs subjected to LPS. Proteins were grouped according to protein groups or origins.

**Figure 5:**
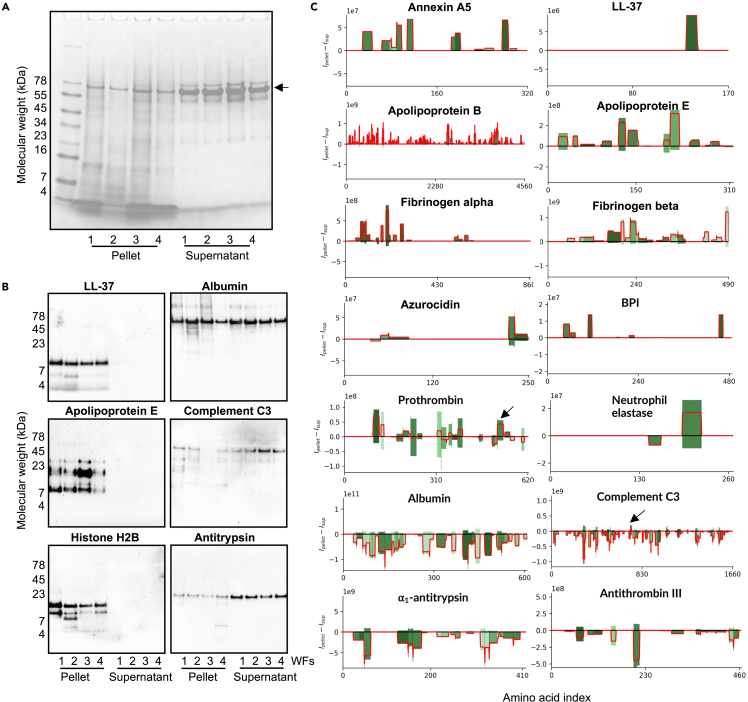
Visualization of proteins and their peptide-fragments (A) SDS-PAGE depicts proteins and peptides in the pellets and supernatants of AWFs subjected to LPS. 30 μg of protein were analyzed. Arrow indicates position of albumin. (B) Western blot analysis of the selected indicated proteins. The images represent an example from at least three independent experiments. 30 μg of protein were analyzed. (C) The x axis in each protein-plot represents the amino acid index and the y axis the summarized intensities in the pellet and supernatant, respectively. The intensities in the pellet go in the positive direction (up) and the supernatant the negative (down). The color of the bars corresponds to the number of peptides overlapping each amino acid index, where darker shades of green correspond to a higher overlap. The color cutoffs are defined by quintiles. The red line shows the difference between the intensities in the pellet and supernatant for each amino acid index—i.e., the difference in bar-heights.

The mean protein MS intensity in the pellets and supernatants was determined, showing an overall correlation between the intensities of proteins in the pellet and supernatants (Figure 4A). However, the observed spread of the intensities indicated that certain proteins were particularly enriched in the pellet. To visualize this, the relative protein abundance in the pellet was calculated, illustrating the overall aggregation tendency of the individual proteins (Figures 4B and 4C). We observed that certain sequences derived from annexins, apolipoproteins, hemoglobins, histones, antimicrobial proteins, and coagulation factors were particularly enriched in the LPS-induced AWF pellets (Figures 4B and 4C). The heat maps illustrating all the identified proteins demonstrate the selectivity of aggregation, defining the “LPS aggregatome” (Figures 4D and S4). To validate the mass spectrometry-based data, we performed SDS-PAGE and Western blot analyses. Initial SDS-PAGE analysis showed that the LPS aggregated material in the pellet contained more low-molecular-weight proteins/peptides when compared with the material in the supernatant (Figure 5A). Albumin, a dominating protein in AWFs, was observed in the region of 65–70 kDa (Figure 5A, arrow), and it was noted that its relative abundance was lower in the LPS pellet (Figure 5A), which is in agreement with the mass spectrometry data (Figure S4). Next, SDS-PAGE followed by Western blot analysis demonstrated a relative increase of LL-37, apolipoprotein E (apoE), and histone H2B in the LPS pellet, whereas a reduction was observed for complement factor C3 and α_1_ antitrypsin (Figure 5B).

In this context, it should be noted that Western blot analysis is a qualitative and semiquantitative method, with signal dynamic range dependent on protein amount loaded onto the gel and exposure time. Therefore, while the reduction in albumin intensity was observed mainly on the Coomassie stained gel (Figure 5A), the absence of significant difference in albumin intensity on the Western blot (Figure 5B) could be attributed to overexposure due to the high abundance of albumin in the samples. Taken together, the SDS-PAGE and Western blot analyses (Figures 5A and 5B) illustrate the selectivity of aggregation, in agreement with the mass spectrometry-based results.

Peptide data can be illustrated in peptigrams showing peptide intensity and position in the protein sequence. Therefore, peptigrams of the peptide sequences identified were generated from a set of selected proteins representing various protein classes and grades enrichment in the LPS aggregatome (Figure 5C). We also wanted to probe whether certain regions from these proteins were enriched, so the peptide intensities were subtracted to visualize the differences between the peptidomes of the LPS pellets and the supernatants (Figure 5C, red line). The results clearly demonstrate the selective enrichment and exclusion of peptide sequences, further illustrating the selectivity of LPS-induced aggregation.

### Effects of AWFs on LPS responses *in vitro* and *in vivo*

**Figure 6:**
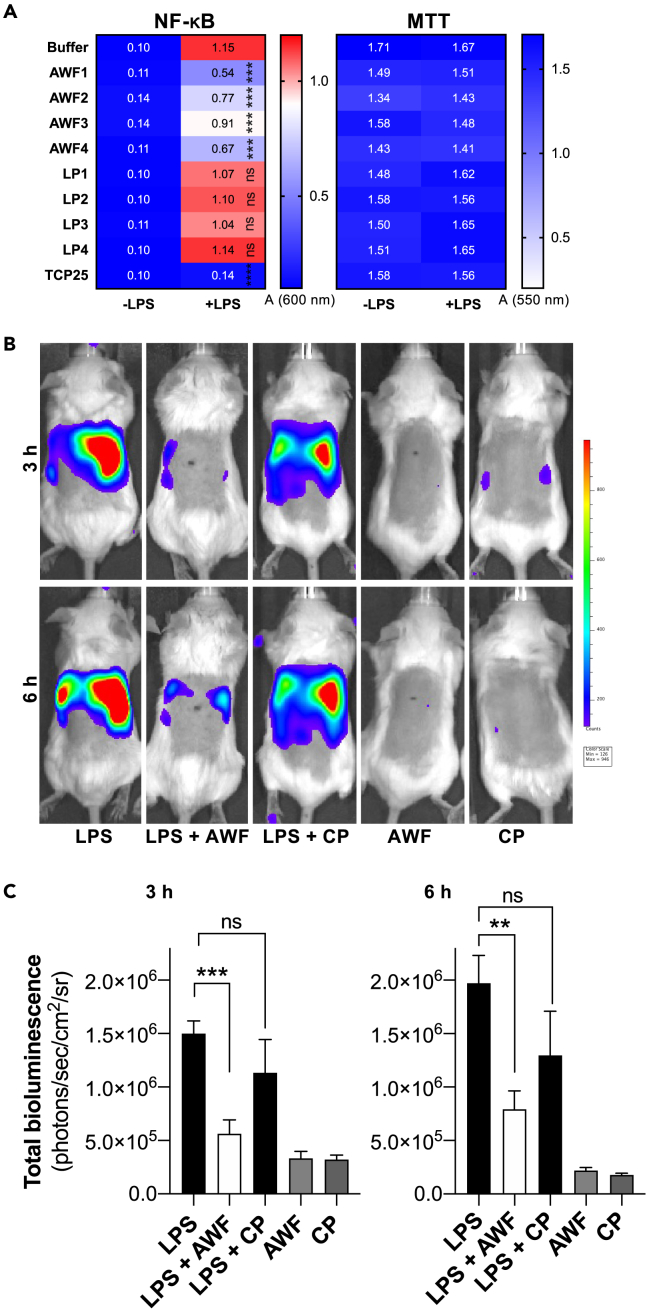
Effects of AWF on LPS-responses *in vivo* (A) THP-1 cells treated with AWFs and LPS exhibited significantly lower activation of NF-κB compared to LPS alone. No reduction of LPS-induced NF-κB activation is seen after addition of lepirudin plasma (LP). MTT viability assay showed no toxic effect of LPS, AWFs, or LPs on THP-1 cells. Statistical analysis was performed using one-way ANOVA with Dunnett’s multiple comparison tests from six independent experiments (n = 6). ∗∗∗ = p ≤ 0.001, ∗∗∗∗ = p ≤ 0.0001, ns = not significant. (B) LPS aggregated by AWF shows reduced NF-κB activation in a mouse model. (C) Mice injected subcutaneously with AWF and LPS show significantly lower NF-κB activation when compared with LPS alone or LPS with citrate plasma (CP). All *in vivo* data are presented as the mean ± SEM (n = 5–6 mice). ∗ = p ≤ 0.05, *p* values were determined using the Mann-Whitney U test.

We explored the functional significance of LPS-induced aggregation. For this, we determined the NF-κB/AP-1 activation in THP-1-XBlue-CD14 reporter monocytes. The results showed that all four AWFs inhibited LPS-induced NF-κB activation relative to the buffer and plasma controls. Lepirudin plasma (LP) was used to avoid scavenging effects on Ca^2+^ in the cell medium. The AWFs did not affect cell viability (Figure 6A). It could be argued that the observed inhibition of NF-κB activation is due to an intrinsic effect of the AWFs, separate from its aggregation-dependent scavenging of LPS. To test this, we conducted the NF-κB activity assay in absence of LPS-induced AWF aggregates, by preincubating the THP-1 cells with LPS, followed by washing and addition of the AWFs. In this setup, the addition of AWFs did not significantly reduce the levels of NF-κB activation, indicating that the LPS scavenging is the main factor underlying the observed reduction of NF-κB activation in this experimental model (Figure S5A). Next, we performed a phagocytosis assay using fluorescence detection to further investigate the interaction between LPS-induced protein aggregates and macrophages. In this assay, we stained the aggregates with Amytracker, allowing us to monitor their uptake by macrophages. Our results showed a significant increase in the uptake of protein aggregates in LPS-challenged AWFs compared to AWFs without LPS treatment (Figure S5B). To confirm the internalization of protein aggregates, we conducted fluorescent microscopy imaging of macrophages that were exposed to the amyloid-forming aggregates stained with Amytracker in AWFs pre-treated with FITC-LPS. This technique enabled us to visualize that the macrophages internalized the protein aggregates, which were both stained with Amytracker and labeled with FITC-labeled LPS (Figure S5C).

In order to study the molecular signaling events affected by LPS-scavenging further, we analyzed the pro-inflammatory cytokines IL-1β and TNF-***α***. For comparison, we used TAK-242, a specific TLR4 inhibitor,^14^ and the peptide TCP-25, which inhibits TLR-signaling via dual LPS and CD-14 interactions.^10^^,^^15^ The results showed that the inhibitory effect on LPS signaling induced by aggregation was comparable to the effects elicited by the two LPS inhibitors alone. Moreover, TAK-242 addition to LPS-AWF aggregates did not yield any statistically significant further reduction of the two cytokines (Figure S6A). The results, as summarized in Figure S6B, demonstrate the significance of AWF-mediated LPS-scavenging and sequestration in inhibiting downstream effects on TLR-4 dependent cytokines, while also depicting the inhibitory action of TAK-242 and TCP-25, both acting downstream of the scavenging mechanism.

Finally, in order to study the physiological role of the AWF-mediated LPS scavenging, we explored whether AWFs could suppress LPS-triggered local inflammation *in vivo.* Using mice reporting NF-κB activation, we found that AWF was able to significantly reduce LPS-induced inflammation when compared with plasma after 3 and 6 h (Figures 6B and 6C). In summary, our study indicated that AWF-mediated LPS aggregation reduces NF-κB activation in monocytes *in vitro* and in experimental mouse models of inflammation, highlighting the significance of AWF-mediated LPS-scavenging and sequestration in inhibiting TLR-4 dependent inflammation.

## Discussion

LPS is a highly proinflammatory substance from Gram-negative bacteria that causes excessive inflammation if not controlled, and we have reported a simple but effective mechanism by which AWF can aggregate and reduce LPS effects. Using NF-κB-reporter mice and IVIS bioimaging, we showed that LPS aggregates in AWF induce a significantly reduced local inflammation when compared with LPS administrated with plasma. Importantly, we observed these aggregates exclusively in porcine wounds challenged with LPS, emphasizing the physiological significance of aggregation in the context of host defense. In the *in vitro* experiments using human AWF, the formed LPS aggregates showed positive staining with the amyloid-specific ThT dye and the Amytracker probe. Similarly, in the *in vivo* porcine wound model, the formed LPS aggregates were stained using the Amytracker probe. These results demonstrated the amyloid nature of the LPS aggregates observed in both the *in vitro* and *in vivo* contexts.

Many of the proteins and peptides enriched in the LPS pellet (Figures 4 and S4), such as hCAP18/LL-37, thrombin, other coagulation factors, apoE, apoB, azurocidin, bacterial permeability increasing protein, annexin A5, eosinophil cationic protein, lysozyme, and histones, are all known for their ability to interact with LPS or LPS-rich bacterial surfaces^2^^,^^9^^,^^13^^,^^16^^,^^17^^,^^18^^,^^19^^,^^20^^,^^21^^,^^22^^,^^23^^,^^24^^,^^25^ (see also Table S1). Interestingly, the capacity to aggregate LPS was observed in human AWF and not in plasma. As AWF contains proteases such as neutrophil elastase and various matrix metalloproteinases, it is therefore plausible that proteolytic events may modify certain holoproteins, enabling their LPS interactions and subsequent aggregation. In agreement with this, SDS-PAGE analysis of the LPS aggregatome showed that it contained low molecular fragments that were not observed in the supernatant (Figure 5A). Moreover, this reasoning is elegantly exemplified by prothrombin and thrombin, which yields 11 kDa C-terminal peptides forming aggregates with LPS upon proteolysis.^12^ Interestingly, a close inspection of the thrombin peptigram shows that peptides derived from this C-terminal region are particularly enriched (Figure 5C, arrow). Intriguingly, a peptide region from an LPS-interacting part of C3a, VFLDCCNYITELRRQ,^8^ was also identified in the LPS pellet, whereas the majority of C3 sequences were found in the supernatant (Figure 5C, arrow).

All these observations indicate a certain degree of LPS selectivity and demonstrate the relevance of the experimental system used. However, it cannot be directly assumed that all enriched proteins in the pellet interact with LPS per se, as proteins may co-aggregate in relation to fibrillar amyloids.^26^^,^^27^ Nevertheless, these results suggest a general response of proteins and peptides, aimed at controlling LPS-induced inflammation, and highlight the LPS aggregatome as a primordial fast-acting defense system in wounds. Activated by proteolysis, this defense system is characterized by direct LPS interactions and likely indirect co-aggregation.

From a pathophysiological perspective, our result on the LPS aggregatome therefore underscores the connection between host defense and amyloidogenesis.^28^^,^^29^^,^^30^^,^^31^^,^^32^ Notably, chronic inflammatory states can disrupt the delicate balance of host defense mechanisms, leading to aberrant aggregation and the progression of amyloid diseases. Indeed, growing evidence has highlighted the intricate relationship between inflammation and amyloid diseases, encompassing conditions such as types of localized and systemic amyloidosis^33^^,^^34^ and neurodegenerative disorders, including Alzheimer’s disease (AD), Parkinson’s disease, and Huntington’s disease.^35^^,^^36^^,^^37^^,^^38^ Proteolysis is a hallmark of inflammation in wounds^39^^,^^40^ and it is therefore worth noting that amyloid aggregates are often composed of proteolytic fragments, derived from the degradation of precursor proteins. These fragments display higher amyloidogenic tendency compared to their precursor proteins,^41^ a finding that provides an additional link to the LPS-induced amyloid formation in the AWFs observed in this report. Interestingly, a persistent inflammatory milieu can exacerbate amyloidosis, leading to a vicious cycle of inflammation and disease progression.^33^^,^^42^ It is therefore notable that both acute and chronic systemic inflammation is associated with an increase in cognitive decline in Alzheimer’s disease.^43^^,^^44^

At the molecular level, it is interesting to note that that proteomic analyses of plaques from patients with AD^45^^,^^46^ and materials from other local and systemic amyloid diseases^47^^,^^48^ present lists of co-aggregated proteins comprising many components found here in the LPS aggregatome, including proteases, coagulation factors, and various apolipoproteins. The similarities could indicate a general response of proteins to the formation of fibrillar assemblies.^27^ Moreover, several components present in the LPS aggregatome have been detected in AD and amyloid diseases (Table S1). For example, apoE is detected together with amyloid-β peptides in brains of AD patients.^49^ Correspondingly, peptides from the receptor and lipid-binding regions of apoE (138–150 and 244–272, respectively), from which synthetic peptides have been shown to exhibit antibacterial and immunomodulatory effects,^50^^,^^51^ were found in the LPS aggregatome. Moreover, serum amyloid P, which is enriched in the LPS induced aggregate, has been reported to interact with bacteria^52^ and found in AD and systemic amyloidoses.^53^ Other proteins with well-described roles in host defense—thrombin,^12^ histones,^6^ and LL-37^2^—are also associated with AD or bind to amyloids^54^^,^^55^^,^^56^ (see also Table S1). All these links therefore lend further support for a connection between host defense and amyloidogenesis.

In conclusion, our study demonstrates that aggregation serves as a host defense mechanism to confine and inhibit endotoxins during wounding and inflammation. We highlight the shared protein and peptide components between the LPS aggregatome and amyloid diseases, emphasizing the connection between host defense, inflammation, and amyloidogenesis. Excessive activation of this natural protective mechanism can lead to the formation of amyloid structures, hence contributing to the pathogenesis of amyloidosis and neurodegenerative disorders. Our findings open avenues for uncovering biomarkers and therapeutic targets for amyloid diseases by elucidating this host defense mechanism’s role in amyloid formation. Furthermore, understanding the underlying mechanisms of dysfunctional and ectopic protein aggregation may pave the way for innovative strategies to mitigate the detrimental effects of amyloid diseases.

### Limitations of the study

While the study identifies the presence of protein aggregates induced by LPS, it does not disclose the underlying molecular mechanisms of how LPS triggers this aggregation. Further mechanistic studies are needed to better understand the processes involved. The mass spectrometry-based proteomic analysis provides valuable information about protein composition, but it might miss low-abundance proteins that could play a role in the aggregation process. Moreover, although the study explores the functional consequences of LPS-induced aggregation in terms of NF-κB activation, cytokine signaling, and phagocytosis, further studies are needed to explore the broader implications of these aggregates on inflammation and amyloidogenesis *in vivo*.

The study primarily focuses on *in vitro* and *in vivo* experiments with AWFs and animal models. While these models provide valuable insights, they might not fully represent the complexity and variability of human physiological responses, potentially limiting the direct clinical relevance of the findings. The study also primarily investigates immediate effects of LPS on AWFs and acute wounds, and the findings may not be directly applicable to situations with chronic infection-inflammation and a long-term sustained LPS release, which can have distinct biochemical characteristics. These long-term effects should be addressed in future studies.

## STAR★Methods

### Key resources table

**Table:** 

REAGENT or RESOURCE	SOURCE	IDENTIFIER
**Antibodies**		

Mouse monoclonal antibody against human apolipoprotein E	Abcam	ab1907
Polyclonal rabbit antibodies against the LL37 epitope	Innovagen AB	http://www.innovagen.com/products
Polyclonal rabbit antibodies against human histone 2b	Abcam	ab1790
Polyclonal rabbit antibodies against human albumin	Dako	A 0001
Polyclonal rabbit antibodies against human α_1_ antitrypsin	Dako	A0012
Anti-mouse HRP-conjugated antibodies	Dako	P0217
Anti-rabbit HRP-conjugated antibodies	Dako	P0260
Polyclonal rabbit antibodies against complement C3	Innovagen AB	http://www.innovagen.com/custom-service-polyclonal-rabbit-igg

**Bacterial and virus strains**		

*Escherichia coli* LPS	Sigma-Aldrich	L2630
Pseudomonas aeruginosa LPS	Sigma-Aldrich	L9143
*Escherichia coli* lipid A	Enzo	AXL-581-200-L002
*Escherichia coli* LPS FITC	LSBio	LS-C71709

**Biological samples**		

Healthy donors	Lund University	https://www.staff.lu.se/research-and-education/research-support/research-ethics-and-animal-testing-ethics
Sterile acute wound fluids	Lund University	https://www.staff.lu.se/research-and-education/research-support/research-ethics-and-animal-testing-ethics

**Chemicals, peptides, and recombinant proteins**		

Uranyl acetate	Sigma-Aldrich	73943
TAK-242	InvivoGen	tlrl-cli95
Quanti-BlueTM	InvivoGen	Rep-qbs
MTT MTT (3-(4,5-dimethylthiazolyl)-2,5-diphenyltetrazolium bromide	Sigma-Aldrich	M5655-1G
Thiflavin T	Sigma-Aldrich	T3516-5G

**Critical commercial assays**		

BCA	Thermo Scientific, USA	Cat#**23227**
LAL	Pierce	A39553
Proinflammatory Panel 1 human kits	Mesoscale	K15049D-2

**Deposited data**		

The mass spectrometry proteomics data	PRIDE [1]	PXD030521

**Experimental models: Cell lines**		

line RAW 264.7	InvivoGen	TIB-/1
NF-κB/AP-1 activation in THP-1-XBlue-CD14 reporter monocytes	ATCC	Thpx-sp

**Experimental models: Organisms/strains**		

Göttingen minipigs	Timeline Bioresearch	https://minipigs.dk/
BALB/c tg(NF-κB-RE-Luc)-Xen reporter male mice	Taconic Biosciences	10499-M

**Software and algorithms**		

ImageJ	Schneider et al.	https://imagej.nih.gov/ij/
UniProt human database release 2019_01	Proteomes · Homo sapiens (Human)	https://www.uniprot.org/proteomes/UP000005640
Zeiss Zen 2.6 [blue editionanti)	ZEISS	https://www.zeiss.com/microscopy/en/products/software/zeiss-zen.html
Python 3.9	Python	https://www.python.org/#:∼:text=Welcome%20to%20Python.org
Living Image 4.0 Software	PerkinElmer	https://www.perkinelmer.com/uk/

**Other**		

carbon-coated grids (copper mesh, 400)	TED PELLA, INC.	01754-7

### Resource availability

#### Lead contact

Further information and requests for resources and reagents should be directed to and will be fulfilled by the lead contact, Jitka Petrlova, (jitka.petrlova@med.lu.se).

#### Materials availability

This study did not generate new unique reagents.

### Experimental model and study participant details

#### Human participants

Sterile acute wound fluids (AWFs) were obtained from surgical drainages after surgery as described previously.^58^ The collection of samples was performed 24–48 hours after surgery. The AWFs were centrifuged, aliquoted, and stored at −20°C. Blood was collected from healthy donors and used for generation of citrated plasma (CP) and lepirudin plasma (LP).

#### Animals

BALB/c tg(NF-κB-RE-Luc)-Xen reporter male mice (Taconic Biosciences) were used for the subcutaneous inflammation experiments and bioimaging. We used a minipig partial-thickness wound model in female Göttingen minipigs (14–16 kg body weight) to study the LPS-induced aggregation *in vivo*. The animals were housed under standard conditions of light and temperature and had free access to standard laboratory chow and water.

#### Cell lines

THP-1 cells (American Type Culture Collection, USA) were cultured in RMPI 1640-GlutaMAX-1 (Gibco, Life Technology ltd., UK). The media was supplemented with 10% (v/v) heat-inactivated FBS (FBSi, Invitrogen, USA) and 1% (v/v) antibiotic-antimycotic solution (AA, Invitrogen) at 37°C in 5% CO_2_. RAW 264.7 cells (American Type Culture Collection, USA) were cultured in DMEM (HyClone, GE Healthcare Life Science, USA) supplemented with 10% (v/v) heat-inactivated FBS (FBSi; Invitrogen, USA) and 1% (v/v) antibiotic-antimycotic solution (AA; Invitrogen).

#### Ethics statement

The use of human wound materials and blood was approved by the Ethics Committee at Lund University (LU 708-01 and LU 509-01; permit no. 657-2008). Informed consent was obtained from all donors. All animal experiments were performed according to the Swedish Animal Welfare Act (SFS 1988:534) and were approved by the Animal Ethics Committee of Malmö/Lund, Sweden (permit numbers M88-91/14, M5934-19, M8871-19). Animals were kept under standard conditions of light and temperature with water provided *ad libitum*.

### Method details

#### Aggregation of AWF proteins by lipopolysaccharide

We incubated four AWFs and four CP samples (100 μl) with increasing concentrations of *Escherichia coli* LPS (0, 50, 100, 200, and 500 μg/ml) (Sigma, USA) in 10 mM Tris for 30 min at pH 7.4°C and 37°C. The samples were spun at 10,000 ×*g* for 5 min, and the pellets and supernatants were collected separately. The pellets were washed with 10 mM Tris pH 7.4 buffer and suspended in 100 μl of 8 M urea. Resuspended pellets and 100× diluted supernatants were analyzed for protein concentration using the bicinchoninic acid (BCA) assay according to the manufacturer’s protocol (Thermo Scientific, USA).

For mass spectrometry, AWFs (500 μl) from four different patients with LPS (50 μg) were incubated at 37°C for 30 min. The mixtures were centrifuged at 10,000 ×*g* for 5 min, and the pellets and supernatants were collected separately. The pellets were washed with 10 mM Tris at pH 7.4 buffer and suspended in a buffer comprising 2% SDS and 50 mM ammonium acetate at pH 6.0. The protein content was determined by the BCA assay.

#### Thioflavin T binding assay

The fluorophore thioflavin T (ThT) (Sigma, USA) was obtained from a 1 mM stock stored in the dark at 4°C. We incubated four AWFs and CPs (20 μl) with or without *E. coli* or *P. aeruginosa* LPS, or *E. coli* lipid A (100 μg/ml) in 10 mM Tris at pH 7.4 for 30 min at 37°C. We added 180 μl of ThT from the stock solution to a final concentration of 100 μM in 10 mM Tris at pH 7.4 and incubated it for 15 min in the dark. We measured ThT fluorescence using a VICTOR3 multilabel plate counter spectrofluorometer (PerkinElmer, USA) at an excitation of 450 nm with excitation and emission slit widths of 10 nm. The signal obtained from LPS in 10 mM Tris at pH 7.4 was defined as the baseline and subtracted from each AWF and CP sample.

#### Transmission electron microscopy – negative stain (TEM)

Aggregates of AWFs were visualized using TEM (Jeol Jem 1230; Jeol, Japan) in combination with negative staining after incubation with LPS from *E. coli* or *P. aeruginosa* (100 μg/ml), or buffer. Images of 10% of AWFs in the presence or absence of LPS (100 μg/ml) were obtained after incubation for 30 min at 37°C. The grids were rendered hydrophilic via glow discharge at low air pressure. Samples were adsorbed onto carbon-coated grids (copper mesh, 400) for 60 s and stained with 7 μl of 2% uranyl acetate for 30 s. For the mounted samples, 10 view fields were examined on the grid (pitch 62 μm) from three independent sample preparations. The CP sample was used in the same experiment as a negative control.

#### Limulus amebocyte lysate (LAL) assay

A LAL assay, performed according to the manufacturer's instructions (Pierce, Thermo Scientific, USA), was used to measure LPS in the pellet (LPS p) and supernatant (LPS s) after addition of LPS. AWFs (20 μl) were mixed with LPS (100 μg/ml) followed by centrifugation (10 min, 10.000 × *g*) as described above. A sample incubated for 30 min, without centrifugation, was used as control. 50 μl of the pellet or supernatant (at a dilution factor of 10^7^) or standard LPS solutions (ranging from 0.1 to 0.0125 pg/ml) was mixed with 50 μl of Amebocyte Lysate Reagent and 50 μl of Reconstitute Chromogenic Substrate. The plate was read at an absorbance of 405 nm after the addition of Stop Solution (25% acetic acid). All samples and solutions were prepared using endotoxin-free water.

#### Minipig partial thickness wound model and WF extraction

Partial thickness wounds in female Göttingen minipigs were induced as described previously.^15^^,^^59^ All procedures were performed by a veterinarian under strict aseptic conditions. The wounding procedures were performed under general anesthesia, and respiratory support was provided with oxygen. Hair on the back was clipped, and the area was thoroughly cleaned with chlorhexidine (MEDI-SCRUB sponge; Rovers, Oss, Netherlands). The back was then shaved and disinfected with chlorhexidine solution (4%) and dried with gauze. Partial-thickness (750-mm deep) wounds (2.5 × 2.5 cm) were made on the back with the help of an electric dermatome (Zimmer).

*E. coli* LPS stock solution was prepared in sterile water (5 mg/ml). A 20-mLl amount of stock solution (100 mg LPS) was mixed in 80 ml of a hydrogel composed of 2% hydroxyethyl cellulose (HEC) and applied directly onto the fresh wound. Blank HEC hydrogel (100 ml) was applied to the control wounds. After 15 minutes, the wounds were covered with a primary foam dressing (Mepilex transfer; Mölnlycke Healthcare, Gothenburg, Sweden), followed by a transparent breathable fixation dressing (Mepore film; Mölnlycke, Gothenburg, Sweden). The film was secured using skin staples (Smi, St. Vith, Belgium). For further protection, the wound was then covered with sterile cotton gauze and finally secured with adhesive tape and flexible self-adhesive bandage (Vet Flex, Kruuse, Denmark).

Animals were housed individually and examined daily. Wound dressings were changed daily, and new hydrogel with LPS or control hydrogel was applied to the wounds.

Polyurethane wound dressings from the porcine partial-thickness wounds were collected aseptically. Dressings were immediately transferred to a 5 ml pre-chilled tube and kept on ice. For the extraction of the porcine wound fluids (PWFs), dressings were soaked in 500 μl of ice-cold 10 mM Tris buffer at pH 7.4 and centrifuged for 5 min (2000 × *g*, 4°C). Extracted PWFs were aliquoted and stored at −80°C until further analysis.

#### Fluorescence microscopy

We used Amytracker 680 (Ebba Biotech, Lund, Sweden) staining to visualize amyloid formation of proteins in human and pig WFs. We incubated WFs (10 μl) with *E. coli* LPS (100 μg/ml) or FITC-LPS (100 μg/ml, from *E. coli*, Sigma Aldrich, USA) for 30 min at 37°C. The samples were subsequently incubated with 50 μl of Amytracker 680 (1000× dilution from the obtained stock solution) in the tube for an additional 30 min of incubation at 37°C. Next, the samples were washed, transferred onto glass slides coated with L-lysine (SIGMA St. Louis, MO, USA), and mounted on microscope slides with fluorescent mounting media (Dako North America, Carpinteria, CA, USA). Ten view fields (1×1 mm) were examined from three independent sample preparations using a Zeiss AxioScope A.1 fluorescence microscope (objectives: Zeiss EC Plan-Neofluar 40×; camera: Zeiss AxioCam MRm; acquisition software: Zeiss Zen 2.6 [blue editionanti). The CP sample was used in the same experiment as a negative control. The size of aggregates was analyzed as the mean of gray value/μm^2^ ± SEM by ImageJ 1.52k, after all the images were converted to 8-bit and the threshold was adjusted.

#### Phagocytosis assay

The macrophage cell line RAW 264.7 (passage 4-7) in DMEM was seeded in 96-well tissue culture plates (8 × 10^4^ cells per well) overnight at 37°C in a 5% CO_2_ atmosphere. AWFs were mixed with and without LPS (100 μg/ml) for 30 minutes at 37°C, which was followed by incubation with Amytracker 680 (1000× dilution from the obtained stock solution, Ebba Biotech, Lund, Sweden) for 30 minutes at the room temperature. The pre-mixed samples (20 μl) were added to the adherent RAW cells. To measure phagocytosis, the samples were incubated with the cells for 1 hour at 37°C and washed twice with DMEM media. We then measured fluorescence using a VICTOR3 Multilabel Plate Counter spectrofluorometer (PerkinElmer, USA) at excitation/emission wavelengths of 620/640 nm. The baseline uptake (of only media) was subtracted from the signal of each sample. For analysis using fluorescence microscopy, RAW 264.7 (passage 4-7) cells were seeded in 24-well tissue culture plates with inserted coverslips (2 × 10^5^ cells per well) overnight at 37°C in a 5% CO_2_ atmosphere. Then cells were treated as described above. After 1 hour incubation at 37°C, cells were stained with 300 ng/ml DAPI (Sigma Aldrich, USA) for 3 min at room temperature, washed with PBS and mounted onto glass slides using a mounting media (Mountant, Permafluor, Thermo Fisher Scientific, KU) and analyzed as described above.

#### NF-κB activity assay

NF-κB/AP-1 activation in THP-1-XBlue-CD14 reporter monocytes was determined after 20–24 h of incubation according to the manufacturer’s protocol (InvivoGen, San Diego, USA). Briefly, 1 × 10^6^ cells/ml in RPMI were seeded in 96-well plates (180 μl) and incubated with pre-mixed AWFs or lepirudin plasma (20 μl) with or without LPS (10 ng/ml) overnight at 37°C in 5% CO_2_ in a total volume of 200 μl. In a separate set of experiments, cells were preincubated with LPS (10 ng/ml) for 1 hour in 96-well plate, washed with PBS (200 μl, two times), followed by addition of AWFs or lepirudin plasma. NF-κB activation was determined after 20 h of incubation according to the manufacturer’s instructions by mixing 20 μl of supernatant with 180 μl of SEAP detection reagent (Quanti-BlueTM, InvivoGen, San Diego, CA, USA). The plates were incubated for 2 hours at 37°C, and the absorbance was measured at 600 nm in a VICTOR3 Multilabel Plate Counter spectrofluorometer. In both experimental setups we used the LPS and CD14 blocking peptide TCP-25 (Ambiopharm, North Augusta, SC, USA) as a positive control for inhibition of LPS-induced NF-κB activation.^10^

#### MTT viability assay

A solution sterile filtered solution of MTT (3-(4,5-dimethylthiazolyl)-2,5-diphenyltetrazolium bromide; 5 mg/mL in PBS; Sigma-Aldrich) was stored in the dark at –20°C until usage. We added 20 μl of MTT solution to the remaining overnight culture of THP-1-XBlue-CD14 reporter monocytes from the NF-κB activity assay in 96-well plates, which were incubated at 37°C for 1 hour. The supernatant was then removed, and the blue formazan product generated in the cells was dissolved by the addition of 100 μl of 100% DMSO to each well. The plates were then gently shaken for 10 min at room temperature to dissolve the precipitates. The absorbance was measured at 550 nm in a VICTOR3 Multilabel Plate Counter spectrofluorometer.

#### Analysis of TNF-α and IL-1β

THP-1-XBlue-CD14 reporter monocytes were incubated with LPS, or LPS-AWFs, and the inhibitors TAK-242 (InvivoGen, USA) (1 μM final concentration) and TCP-25^10^ (2 μM final concentration). Following incubation for 20-24 hours, the supernatants were collected and TNF-α and IL-1β analysis was performed using the MESO QuickPlex SQ 120MM instrument (Mesoscale, USA) according to the manufacturer's protocol. In summary, we added 50 μl of undiluted supernatants and calibrators into the wells of a V-PLEX plate (Mesoscale, USA). The plate was then incubated at room temperature for two hours. Following incubation, the plate was washed with PBS-T, and 25 μl of each detection antibody was added. The detection antibody was diluted 50 times and added to the plate wells. After washing, the read buffer was added, and the plate was analyzed. Values were determined relative a standard according to the manufacturer's protocol.

#### Mouse inflammation model

BALB/c tg(NF-κB-RE-Luc)-Xen reporter mice (Taconic, 10–12-weeks old) were used to study the effects of AWF1 on inflammation (200 μl) after subcutaneous co-treatment with LPS (*E. coli,* 25 μg). The samples were pre-incubated for 30 min at 37°C before injection. The dorsa of the mice were carefully shaved and cleaned. The mice were anesthetized with isoflurane, and 200 μl of the sample was injected subcutaneously. The animals were immediately transferred to individually ventilated cages (IVC) and imaged 3 and 6 h later.

We used an *In Vivo* Imaging System (IVIS) for the determination of NF-κB activation, which plays a key role in the regulation of immune responses during infection. Bioluminescence from the mice was detected and quantified using Living Image 4.0 Software. The software automatically calibrates photon radiance to NIST standards, enabling reproducible, quantitative results by incorporating instrument calibration, background subtraction, and image processing algorithms (PerkinElmer, USA). Mice were administrated 100 μl of D-Luciferin intraperitoneally 15 minutes before IVIS imaging (150 mg/kg body weight).^15^

#### LC-MS/MS analysis

The protein pellets and supernatants (40 μg total protein) from the WFs aggregated with LPS were separated using sodium dodecyl sulfate-polyacrylamide gel electrophoresis (SDS-PAGE; 5% polyacrylamide stacking gel with 12% polyacrylamide separating gel). Electrophoretic separation was performed at 80 V for 20 min and then at 100 V for another 80 min. The gel was then fixed and stained with Coomassie brilliant blue. Next, each sample lane was sliced separately, cut into small pieces of approximately 1 mm^2^, and subjected to complete destaining. After destaining, the gel pieces were reduced with 10 mM DTT, alkylated using 55 mM iodoacetamide, dehydrated with 100% acetonitrile, and then subjected to overnight digestion at 37°C with sequencing-grade modified trypsin (Promega, Madison, WI, USA). The peptides were extracted, vacuum dried, and reconstituted in 0.1% formic acid for LC-MS/MS analysis.

Peptides from four biological replicates and their technical duplicates were separated and analyzed in an LC-MS/MS system comprising of a Dionex Ultimate 3000 RSLC nano-HPLC system, coupled to an online Q-Exactive mass spectrometer (Thermo-Fisher Scientific, USA). Five microliters of each sample were injected into an acclaim peptide trap column via the auto-sampler of the Dionex RSLC nano-HPLC system. The mobile phase A (0.1% FA in 5% acetonitrile) and mobile phase B (0.1% FA in acetonitrile) were used to establish a 60-minute gradient, and the flow rate was maintained at 300 nl/ml. Peptides were analyzed using a Dionex EASY-spray column (PepMap® C18, 3um, 100 A) using an EASY nanospray source. The electrospray potential was set at 1.6 kV.

A full MS scan in the range of 350-1600 m/z was acquired at a resolution of 70,000 at m/z 200 with a maximum ion accumulation time of 100 ms. The dynamic exclusion was set to 30 seconds. The resolution for MS/MS spectra was set to 35,000 at m/z 200. The AGC setting was 1E6 for the full MS scan and 2E5 for the MS2 scan. The 10 most intense ions above a count threshold of 1000 were chosen for higher energy collision dissociation (HCD) fragmentation. The maximum ion accumulation time was 120 ms, and an isolation width of 2 Da was used for the MS2 scan. Single and unassigned charged ions were excluded from MS/MS. For HCD, the normalized collision energy was set to 28, and the underfill ratio was defined as 0.1%.

#### Mass spectrometry data analysis

Acquired data were processed using Proteome Discoverer (PD) v1.4 (Thermo Scientific, San Jose, CA, USA) with deisotope and deconvolution in MS/MS. The raw files were directly imported into PD and further processed using the designed workflow. Briefly, this workflow includes five processing nodes numbered from 0 to 5. Node 0 is named “spectrum file” and allows selecting raw files, while node 1 is labeled as “spectrum selector” and extracts, deisotopes, and deconvolutes the spectra within a retention time window and precursor ion mass window.

Node 2 was an MS spectrum processor, while node 3 selected the search engine SequestHT, and node 4 used Mascot with database search parameters. The parameter settings were the following: enzyme: trypsin, maximum miss cleavage: 2, minimum peptide length: 6, maximum peptide length: 144, precursor mass tolerance: 10 ppm, fragment mass tolerance: 0.02 Da, dynamic modification: deamidation of Q and N. Node 5 was called “percolator,” and the target FDR (strict) was set as 0.01, while the target FDR (relaxed) was set as 0.05. A database search was performed against the UniProt human database release 2017_02.

For peptidomic analyses, peptide files generated after searching the raw data with PEAKS X were used. For this search, the same modifications and mass tolerances as above were used except that we applied the “no enzyme” option, allowing unspecific cleavages. A database search was performed against the UniProt human database release 2019_01 using 1% FDR and a minimum of two peptides per protein. The obtained peptide/protein lists were exported to Microsoft Excel and analyzed using algorithms in Python 3.9.

The mean protein intensities between the pellets and supernatants collected from all four AWFs were calculated and used to visualize and determine the specificity of LPS-triggered protein aggregation. To visualize the relative discrepancies in protein abundancy between pellet and supernatant, the intensity fraction was defined as follows:

$$Relative\;protein\;abundance\;in\;pellet=\frac{I_{pellet}}{I_{pellet}+I_{supernatant}}$$

$$Relative\;protein\;abundance\;in\;supernatant=\frac{I_{supernatant}}{I_{pellet}+I_{supernatant}}$$

These abundances were calculated for each AWF separately and visualized in heat maps.

The peptidomic content of the pellet and supernatant were visualized using a common convention where the amino acid indexes of proteins are plotted against the abundance of overlapping peptides covering the given index. For a given protein, the abundance *a*_*i*_ of an amino acid at position *i* is the sum of peptide intensities for all peptides that overlap at *i*. The amino acid abundances were then summarized for samples in the pellet and supernatant, resulting in two vectors with the abundance for each amino acid index for each protein.

#### SDS-PAGE and western blot

We separated 30 μg of proteins from pellets and supernatants of each AWF using sodium dodecyl sulfate-polyacrylamide gel electrophoresis (Tricine; 10% polyacrylamide stacking gel with 20% polyacrylamide separating gel, Novex by Life Technologies, Carlsbad, CA, USA). Electrophoretic separation was performed at 100 V for 110 min. The gels were fixed and stained with Blue Safe Protein Stain (Thermo Scientific, Rockford, IL, USA) or transferred to a PVDF membrane using a Trans-Blot Turbo system (Bio-Rad, Hercules, CA, USA). The following primary antibodies were used for the western blot experiments: mouse monoclonal antibody against human apolipoprotein E (dilution 1:1000; Abcam, Cambridge, UK), and polyclonal rabbit antibodies against the LL37 epitope (diluted 1:1000; Innovagen AB, Lund, Sweden), human histone 2b (diluted 1:1000; Abcam, Cambridge, UK), human albumin (diluted 1:1000; Dako, Carpinteria, CA, USA), complement C3 (diluted 1:1000; Innovagen AB, Lund, Sweden), and human α_1_ antitrypsin (diluted 1:1000; Dako, Carpinteria, CA, USA). Next, anti-rabbit or anti-mouse HRP-conjugated antibodies (1:2000; Dako, Carpinteria, CA, USA) were used for detection of the primary antibodies. The proteins and peptides were visualized using chemiluminescent substrates (Thermo Scientific, Rockford, IL, USA) using a ChemiDoc MP imaging system (Bio-Rad, Hercules, CA, USA).

### Quantification and Statistical analysis

The graphs of BCA, ThT, and NF-κB activity assays, phagocytosis assay, LAL assay, cytokine analysis are presented as means ± SD from at least three independent experiments. We assessed differences in these assays using a one-way ANOVA with Dunnett’s multiple comparison tests. Data were analyzed using GraphPad Prism (ααGraphPad Software, Inc., USA) and Python 3.9. P-values less than 0.05 were considered statistically significant (∗ P < 0.05, ∗∗ P < 0.01, ∗∗∗ P < 0.001, and ∗∗∗∗ P < 0.0001).

## Data Availability

• All mass spectrometry proteomics datasets have been deposited at the ProteomeXchange Consortium via the PRIDE and is publicly available as of the date of publication. See key resources table. • This paper does not report original code. • Any additional information required to reanalyze the data reported in this paper is available from the lead contact upon request.
