# Supplementary material for: Selective protein aggregation confines and inhibits endotoxins in wounds: Linking host defense to amyloid formation
Source: iScience. 2023 Sep 19;26(10):107951. doi: 10.1016/j.isci.2023.107951 (PMC10561040; doi:10.1016/j.isci.2023.107951)
Supplement: Document S1. Figures S1–S6 and Table S1 [file mmc1.pdf]

iScience, Volume *26*

## Supplemental information

# Selective protein aggregation confines and inhibits endotoxins in wounds: Linking host defense to amyloid formation

**Jitka Petrlova, Erik Hartman, Ganna Petruk, Jeremy Chun Hwee Lim, Sunil Shankar Adav, Sven Kjellström, Manoj Puthia, and Artur Schmidtchen**

**Supplemental information**

**Table S1**

**Proteins aggregating in the presence of LPS and potential links to LPS binding, antimicrobial activity, amyloid formation, or Alzheimer’s disease (AD), related to Figure 4.** Proteins/peptides are shown in falling aggregation index order. Note that the list shows associations, and certain components, such as apolipoproteins or serum amyloids, may be involved in pathogenesis or, in the case of lysozyme, have possible protective effects on amyloid formation or AD development. It should also be noted that amyloidogenesis is involved in AD pathogenesis, and hence the division in “amyloid” and “AD” in the list merely reflects whether the published work is more focused on amyloid formation or disease-oriented aspects of Alzheimer's disease.

| **Protein** | **LPS-interaction** | **Antimicrobial** | **Amyloid** | **AD** |
|---|---|---|---|---|
| Bactericidal/permeability increasing protein | (1,2) | (1) | | |
| Annexin A5 | (3) | | (4) | (4,5) |
| CAMP (LL-37) | (6-8) | (8) | (9,10) | (11,12) |
| Eosinophil cationic protein | (13) | (14) | (14,15) | (16) |
| Fibrinogen alpha | | | (17) | (18,19) |
| Fibrinogen beta | | (20) | | (19) |
| Apolipoprotein B | (21) | (22) | (23) | (24) |
| Apolipoprotein E | (25,26) | (27-29) | (23,30,31) | (32-34) |
| Histone H2B | (35) | (36) | (37) | (38,39) |
| Histone H4 | (35) | (40) | (41) | (39) |
| Azurocidin | (42) | (43,44) | | (45) |
| Lysozyme C | (46) | | (17,47,48) | (49) |
| Neutrophil elastase | | | (50) | (12) |
| Hemoglobin alpha and beta chain | (51) | (52-54) | (55,56) | (57-60) |
| Thrombin | (61) | (62,63) | (64,65) | (66-68) |
| Serum amyloid protein | (69,70) | (70,71) | (72-74) | (72,73,75) |
| NGAL | (76) | (76) | (77) | (78) |
| Histone H3 | (36) | (35,79) | (41) | (80) |
| Apolipoprotein A1 | (81,82) | (83) | (84-86) | (87-89) |

1. Elsbach, P., and Weiss, J. (1998) Role of the bactericidal/permeability-increasing protein in host defence. *Curr Opin Immunol* **10**, 45-49
2. Wilde, C. G., Seilhamer, J. J., McGrogan, M., Ashton, N., Snable, J. L., Lane, J. C., Leong, S. R., Thornton, M. B., Miller, K. L., Scott, R. W., and et al. (1994) Bactericidal/permeability-increasing protein and lipopolysaccharide (LPS)-binding protein. LPS binding properties and effects on LPS-mediated cell activation. *J Biol Chem* **269**, 17411-17416
3. Rand, J. H., Wu, X. X., Lin, E. Y., Griffel, A., Gialanella, P., and McKitrick, J. C. (2012) Annexin A5 binds to lipopolysaccharide and reduces its endotoxin activity. *mBio* **3**
4. Bedrood, S., Jayasinghe, S., Sieburth, D., Chen, M., Erbel, S., Butler, P. C., Langen, R., and Ritzel, R. A. (2009) Annexin A5 directly interacts with amyloidogenic proteins and reduces their toxicity. *Biochemistry* **48**, 10568-10576
5. Sohma, H., Imai, S., Takei, N., Honda, H., Matsumoto, K., Utsumi, K., Matsuki, K., Hashimoto, E., Saito, T., and Kokai, Y. (2013) Evaluation of annexin A5 as a biomarker for Alzheimer's disease and dementia with lewy bodies. *Front Aging Neurosci* **5**, 15
6. Ong, P. Y., Ohtake, T., Brandt, C., Strickland, I., Boguniewicz, M., Ganz, T., Gallo, R. L., and Leung, D. Y. (2002) Endogenous antimicrobial peptides and skin infections in atopic dermatitis. *N Engl J Med* **347**, 1151-1160
7. Durr, U. H., Sudheendra, U. S., and Ramamoorthy, A. (2006) LL-37, the only human member of the cathelicidin family of antimicrobial peptides. *Biochim Biophys Acta* **1758**, 1408-1425
8. Wang, G., Mishra, B., Epand, R. F., and Epand, R. M. (2014) High-quality 3D structures shine light on antibacterial, anti-biofilm and antiviral activities of human cathelicidin LL-37 and its fragments. *Biochim Biophys Acta* **1838**, 2160-2172
9. Sood, R., Domanov, Y., Pietiainen, M., Kontinen, V. P., and Kinnunen, P. K. (2008) Binding of LL-37 to model biomembranes: insight into target vs host cell recognition. *Biochim Biophys Acta* **1778**, 983-996
10. Lee, E. Y., Srinivasan, Y., de Anda, J., Nicastro, L. K., Tukel, C., and Wong, G. C. L. (2020) Functional Reciprocity of Amyloids and Antimicrobial Peptides: Rethinking the Role of Supramolecular Assembly in Host Defense, Immune Activation, and Inflammation. *Front Immunol* **11**, 1629
11. De Lorenzi, E., Chiari, M., Colombo, R., Cretich, M., Sola, L., Vanna, R., Gagni, P., Bisceglia, F., Morasso, C., Lin, J. S., Lee, M., McGeer, P. L., and Barron, A. E. (2017) Evidence that the Human Innate Immune Peptide LL-37 may be a Binding Partner of Amyloid-beta and Inhibitor of Fibril Assembly. *J Alzheimers Dis* **59**, 1213-1226
12. Stock, A. J., Kasus-Jacobi, A., and Pereira, H. A. (2018) The role of neutrophil granule proteins in neuroinflammation and Alzheimer's disease. *J Neuroinflammation* **15**, 240
13. Pulido, D., Garcia-Mayoral, M. F., Moussaoui, M., Velazquez, D., Torrent, M., Bruix, M., and Boix, E. (2016) Structural basis for endotoxin neutralization by the eosinophil cationic protein. *FEBS J* **283**, 4176-4191
14. Torrent, M., Pulido, D., Nogues, M. V., and Boix, E. (2012) Exploring new biological functions of amyloids: bacteria cell agglutination mediated by host protein aggregation. *PLoS pathogens* **8**, e1003005

15. Torrent, M., Odorizzi, F., Nogues, M. V., and Boix, E. (2010) Eosinophil cationic protein aggregation: identification of an N-terminus amyloid prone region. *Biomacromolecules* **11**, 1983-1990
16. Navarro, S., Boix, E., Cuchillo, C. M., and Nogues, M. V. (2010) Eosinophil-induced neurotoxicity: the role of eosinophil cationic protein/RNase 3. *J Neuroimmunol* **227**, 60-70
17. Biza, K. V., Nastou, K. C., Tsiolaki, P. L., Mastrokalou, C. V., Hamodrakas, S. J., and Iconomidou, V. A. (2017) The amyloid interactome: Exploring protein aggregation. *PLoS One* **12**, e0173163
18. Benson, M. D., Liepnieks, J., Uemichi, T., Wheeler, G., and Correa, R. (1993) Hereditary renal amyloidosis associated with a mutant fibrinogen alpha-chain. *Nat Genet* **3**, 252-255
19. Kiddle, S. J., Thambisetty, M., Simmons, A., Riddoch-Contreras, J., Hye, A., Westman, E., Pike, I., Ward, M., Johnston, C., Lupton, M. K., Lunnon, K., Soininen, H., Kloszewska, I., Tsolaki, M., Vellas, B., Mecocci, P., Lovestone, S., Newhouse, S., Dobson, R., and Alzheimers Disease Neuroimaging, I. (2012) Plasma based markers of [11C] PiB-PET brain amyloid burden. *PLoS One* **7**, e44260
20. Pahlman, L. I., Morgelin, M., Kasetty, G., Olin, A. I., Schmidtchen, A., and Herwald, H. (2013) Antimicrobial activity of fibrinogen and fibrinogen-derived peptides--a novel link between coagulation and innate immunity. *Thromb Haemost* **109**, 930-939
21. Vreugdenhil, A. C., Snoek, A. M., van 't Veer, C., Greve, J. W., and Buurman, W. A. (2001) LPS-binding protein circulates in association with apoB-containing lipoproteins and enhances endotoxin-LDL/VLDL interaction. *J Clin Invest* **107**, 225-234
22. Gaglione, R., Cesaro, A., Dell'Olmo, E., Della Ventura, B., Casillo, A., Di Girolamo, R., Velotta, R., Notomista, E., Veldhuizen, E. J. A., Corsaro, M. M., De Rosa, C., and Arciello, A. (2019) Effects of human antimicrobial cryptides identified in apolipoprotein B depend on specific features of bacterial strains. *Sci Rep* **9**, 6728
23. Mullins, R. F., Russell, S. R., Anderson, D. H., and Hageman, G. S. (2000) Drusen associated with aging and age-related macular degeneration contain proteins common to extracellular deposits associated with atherosclerosis, elastosis, amyloidosis, and dense deposit disease. *Faseb J* **14**, 835-846
24. Namba, Y., Tsuchiya, H., and Ikeda, K. (1992) Apolipoprotein B immunoreactivity in senile plaque and vascular amyloids and neurofibrillary tangles in the brains of patients with Alzheimer's disease. *Neurosci Lett* **134**, 264-266
25. Petruk, G., Elven, M., Hartman, E., Davoudi, M., Schmidtchen, A., Puthia, M., and Petrlova, J. (2021) The role of full-length apolipoprotein E in clearance of Gram-negative bacteria and their endotoxins. *J Lipid Res*, 100086
26. Puthia, M., Marzinek, J. K., Petruk, G., Erturk Bergdahl, G., Bond, P. J., and Petrlova, J. (2022) Antibacterial and Anti-Inflammatory Effects of Apolipoprotein E. *Biomedicines* **10**
27. Wang, C. Q., Yang, C. S., Yang, Y., Pan, F., He, L. Y., and Wang, A. M. (2013) An apolipoprotein E mimetic peptide with activities against multidrug-resistant bacteria and immunomodulatory effects. *J Pept Sci* **19**, 745-750
28. Azuma, M., Kojimab, T., Yokoyama, I., Tajiri, H., Yoshikawa, K., Saga, S., and Del Carpio, C. A. (2000) A synthetic peptide of human apoprotein E with antibacterial activity. *Peptides* **21**, 327-330
29. Zanfardino, A., Bosso, A., Gallo, G., Pistorio, V., Di Napoli, M., Gaglione, R., Dell'Olmo, E., Varcamonti, M., Notomista, E., Arciello, A., and Pizzo, E. (2018)

Human apolipoprotein E as a reservoir of cryptic bioactive peptides: The case of ApoE 133-167. *J Pept Sci* **24**, e3095
30. Furumoto, H., Hashimoto, Y., Muto, M., Shimizu, T., and Nakamura, K. (2002) Apolipoprotein E4 is associated with primary localized cutaneous amyloidosis. *J Invest Dermatol* **119**, 532-533
31. Furumoto, H., Shimizu, T., Asagami, C., Muto, M., Takahashi, M., Hoshii, Y., Ishihara, T., and Nakamura, K. (1998) Apolipoprotein E is present in primary localized cutaneous amyloidosis. *J Invest Dermatol* **111**, 417-421
32. Cedazo-Minguez, A., and Cowburn, R. F. (2001) Apolipoprotein E: a major piece in the Alzheimer's disease puzzle. *J Cell Mol Med* **5**, 254-266
33. Yamazaki, Y., Zhao, N., Caulfield, T. R., Liu, C. C., and Bu, G. (2019) Apolipoprotein E and Alzheimer disease: pathobiology and targeting strategies. *Nat Rev Neurol* **15**, 501-518
34. Husain, M. A., Laurent, B., and Plourde, M. (2021) APOE and Alzheimer's Disease: From Lipid Transport to Physiopathology and Therapeutics. *Front Neurosci* **15**, 630502
35. Morita, S., Tagai, C., Shiraishi, T., Miyaji, K., and Iwamuro, S. (2013) Differential mode of antimicrobial actions of arginine-rich and lysine-rich histones against Gram-positive Staphylococcus aureus. *Peptides* **48**, 75-82
36. Kawasaki, H., and Iwamuro, S. (2008) Potential roles of histones in host defense as antimicrobial agents. *Infect Disord Drug Targets* **8**, 195-205
37. Du Clos, T. W. (1996) The interaction of C-reactive protein and serum amyloid P component with nuclear antigens. *Mol Biol Rep* **23**, 253-260
38. Zafar, S., Shafiq, M., Younas, N., Schmitz, M., Ferrer, I., and Zerr, I. (2017) Prion Protein Interactome: Identifying Novel Targets in Slowly and Rapidly Progressive Forms of Alzheimer's Disease. *J Alzheimers Dis* **59**, 265-275
39. Lu, X., Wang, L., Yu, C., Yu, D., and Yu, G. (2015) Histone Acetylation Modifiers in the Pathogenesis of Alzheimer's Disease. *Front Cell Neurosci* **9**, 226
40. Lee, D. Y., Huang, C. M., Nakatsuji, T., Thiboutot, D., Kang, S. A., Monestier, M., and Gallo, R. L. (2009) Histone H4 is a major component of the antimicrobial action of human sebocytes. *J Invest Dermatol* **129**, 2489-2496
41. Munishkina, L. A., Fink, A. L., and Uversky, V. N. (2004) Conformational prerequisites for formation of amyloid fibrils from histones. *J Mol Biol* **342**, 1305-1324
42. Heinzelmann, M., Mercer-Jones, M. A., Flodgaard, H., and Miller, F. N. (1998) Heparin-binding protein (CAP37) is internalized in monocytes and increases LPS-induced monocyte activation. *Journal of Immunology* **160**, 5530-5536
43. Shafer, W. M., Martin, L. E., and Spitznagel, J. K. (1984) Cationic antimicrobial proteins isolated from human neutrophil granulocytes in the presence of diisopropyl fluorophosphate. *Infect Immun* **45**, 29-35
44. Pereira, H. A., Erdem, I., Pohl, J., and Spitznagel, J. K. (1993) Synthetic bactericidal peptide based on CAP37: a 37-kDa human neutrophil granule-associated cationic antimicrobial protein chemotactic for monocytes. *Proc Natl Acad Sci U S A* **90**, 4733-4737
45. Pereira, H. A., Kumar, P., and Grammas, P. (1996) Expression of CAP37, a novel inflammatory mediator, in Alzheimer's disease. *Neurobiol Aging* **17**, 753-759
46. Ohno, N., and Morrison, D. C. (1989) Lipopolysaccharide interactions with lysozyme differentially affect lipopolysaccharide immunostimulatory activity. *Eur J Biochem* **186**, 629-636

47. Booth, D. R., Sunde, M., Bellotti, V., Robinson, C. V., Hutchinson, W. L., Fraser, P. E., Hawkins, P. N., Dobson, C. M., Radford, S. E., Blake, C. C., and Pepys, M. B. (1997) Instability, unfolding and aggregation of human lysozyme variants underlying amyloid fibrillogenesis. *Nature* **385**, 787-793
48. Helmfors, L., Boman, A., Civitelli, L., Nath, S., Sandin, L., Janefjord, C., McCann, H., Zetterberg, H., Blennow, K., Halliday, G., Brorsson, A. C., and Kagedal, K. (2015) Protective properties of lysozyme on beta-amyloid pathology: implications for Alzheimer disease. *Neurobiol Dis* **83**, 122-133
49. Sandin, L., Bergkvist, L., Nath, S., Kielkopf, C., Janefjord, C., Helmfors, L., Zetterberg, H., Blennow, K., Li, H., Nilsberth, C., Garner, B., Brorsson, A. C., and Kagedal, K. (2016) Beneficial effects of increased lysozyme levels in Alzheimer's disease modelled in Drosophila melanogaster. *FEBS J* **283**, 3508-3522
50. Stone, P. J., Campistol, J. M., Abraham, C. R., Rodgers, O., Shirahama, T., and Skinner, M. (1993) Neutrophil proteases associated with amyloid fibrils. *Biochem Biophys Res Commun* **197**, 130-136
51. Bahl, N., Du, R., Winarsih, I., Ho, B., Tucker-Kellogg, L., Tidor, B., and Ding, J. L. (2011) Delineation of lipopolysaccharide (LPS)-binding sites on hemoglobin: from in silico predictions to biophysical characterization. *J Biol Chem* **286**, 37793-37803
52. Parish, C. A., Jiang, H., Tokiwa, Y., Berova, N., Nakanishi, K., McCabe, D., Zuckerman, W., Xia, M. M., and Gabay, J. E. (2001) Broad-spectrum antimicrobial activity of hemoglobin. *Bioorg Med Chem* **9**, 377-382
53. Sheshadri, P., and Abraham, J. (2012) Antimicrobial properties of hemoglobin. *Immunopharmacol Immunotoxicol* **34**, 896-900
54. Deng, L. X., Pan, X. L., Wang, Y., Wang, L. L., Zhou, X. E., Li, M., Feng, Y., Wu, Q., Wang, B. Y., and Huang, N. (2009) Hemoglobin and its derived peptides may play a role in the antibacterial mechanism of the vagina. *Hum Reprod* **24**, 211-218
55. Iram, A., and Naeem, A. (2013) Detection and analysis of protofibrils and fibrils of hemoglobin: implications for the pathogenesis and cure of heme loss related maladies. *Arch Biochem Biophys* **533**, 69-78
56. Heuschkel, M. A., Skenteris, N. T., Hutcheson, J. D., van der Valk, D. D., Bremer, J., Goody, P., Hjortnaes, J., Jansen, F., Bouten, C. V. C., van den Bogaerdt, A., Matic, L., Marx, N., and Goettsch, C. (2020) Integrative Multi-Omics Analysis in Calcific Aortic Valve Disease Reveals a Link to the Formation of Amyloid-Like Deposits. *Cells* **9**
57. Arioz, B. I., Tufekci, K. U., Olcum, M., Durur, D. Y., Akarlar, B. A., Ozlu, N., Bagriyanik, H. A., Keskinoglu, P., Yener, G., and Genc, S. (2021) Proteome profiling of neuron-derived exosomes in Alzheimer's disease reveals hemoglobin as a potential biomarker. *Neurosci Lett* **755**, 135914
58. Kim, J. W., Byun, M. S., Yi, D., Lee, J. H., Jeon, S. Y., Ko, K., Joung, H., Jung, G., Lee, J. Y., Sohn, C. H., Lee, Y. S., Kim, Y. K., and Lee, D. Y. (2021) Blood Hemoglobin, in-vivo Alzheimer Pathologies, and Cognitive Impairment: A Cross-Sectional Study. *Front Aging Neurosci* **13**, 625511
59. Yoo, S. H., Woo, S. W., Shin, M. J., Yoon, J. A., Shin, Y. I., and Hong, K. S. (2020) Diagnosis of Mild Cognitive Impairment Using Cognitive Tasks: A Functional Near-Infrared Spectroscopy Study. *Curr Alzheimer Res* **17**, 1145-1160
60. Gattas, B. S., Ibetoh, C. N., Stratulat, E., Liu, F., Wuni, G. Y., Bahuva, R., Shafiq, M. A., and Gordon, D. K. (2020) The Impact of Low Hemoglobin Levels on Cognitive Brain Functions. *Cureus* **12**, e11378
61. Kalle, M., Papareddy, P., Kasetty, G., Morgelin, M., van der Plas, M. J., Rydengard, V., Malmsten, M., Albiger, B., and Schmidtchen, A. (2012) Host defense peptides of

thrombin modulate inflammation and coagulation in endotoxin-mediated shock and Pseudomonas aeruginosa sepsis. *PLoS One* **7**, e51313
62. Tang, Y. Q., Yeaman, M. R., and Selsted, M. E. (2002) Antimicrobial peptides from human platelets. *Infect Immun* **70**, 6524-6533
63. Papareddy, P., Rydengard, V., Pasupuleti, M., Walse, B., Morgelin, M., Chalupka, A., Malmsten, M., and Schmidtchen, A. (2010) Proteolysis of human thrombin generates novel host defense peptides. *PLoS pathogens* **6**, e1000857
64. Petrlova, J., Hansen, F. C., van der Plas, M. J. A., Huber, R. G., Morgelin, M., Malmsten, M., Bond, P. J., and Schmidtchen, A. (2017) Aggregation of thrombin-derived C-terminal fragments as a previously undisclosed host defense mechanism. *Proc Natl Acad Sci U S A* **114**, E4213-E4222
65. Gastineau, D. A., Gertz, M. A., Daniels, T. M., Kyle, R. A., and Bowie, E. J. (1991) Inhibitor of the thrombin time in systemic amyloidosis: a common coagulation abnormality. *Blood* **77**, 2637-2640
66. Akiyama, H., Ikeda, K., Kondo, H., and McGeer, P. L. (1992) Thrombin accumulation in brains of patients with Alzheimer's disease. *Neurosci Lett* **146**, 152-154
67. Arai, T., Miklossy, J., Klegeris, A., Guo, J. P., and McGeer, P. L. (2006) Thrombin and prothrombin are expressed by neurons and glial cells and accumulate in neurofibrillary tangles in Alzheimer disease brain. *J Neuropathol Exp Neurol* **65**, 19-25
68. Zamolodchikov, D., Renne, T., and Strickland, S. (2016) The Alzheimer's disease peptide beta-amyloid promotes thrombin generation through activation of coagulation factor XII. *Journal of Thrombosis and Haemostasis* **14**, 995-1007
69. de Haas, C. J., van Leeuwen, E. M., van Bommel, T., Verhoef, J., van Kessel, K. P., and van Strijp, J. A. (2000) Serum amyloid P component bound to gram-negative bacteria prevents lipopolysaccharide-mediated classical pathway complement activation. *Infect Immun* **68**, 1753-1759
70. Noursadeghi, M., Bickerstaff, M. C., Gallimore, J. R., Herbert, J., Cohen, J., and Pepys, M. B. (2000) Role of serum amyloid P component in bacterial infection: protection of the host or protection of the pathogen. *Proc Natl Acad Sci U S A* **97**, 14584-14589
71. Hind, C. R., Collins, P. M., Baltz, M. L., and Pepys, M. B. (1985) Human serum amyloid P component, a circulating lectin with specificity for the cyclic 4,6-pyruvate acetal of galactose. Interactions with various bacteria. *Biochem J* **225**, 107-111
72. Tennent, G. A., Lovat, L. B., and Pepys, M. B. (1995) Serum amyloid P component prevents proteolysis of the amyloid fibrils of Alzheimer disease and systemic amyloidosis. *Proc Natl Acad Sci U S A* **92**, 4299-4303
73. Coria, F., Castano, E., Prelli, F., Larrondo-Lillo, M., van Duinen, S., Shelanski, M. L., and Frangione, B. (1988) Isolation and characterization of amyloid P component from Alzheimer's disease and other types of cerebral amyloidosis. *Lab Invest* **58**, 454-458
74. Botto, M., Hawkins, P. N., Bickerstaff, M. C. M., Herbert, J., Bygrave, A. E., McBride, A., Hutchinson, W. L., Tennent, G. A., Walport, M. J., and Pepys, M. B. (1997) Amyloid deposition is delayed in mice with targeted deletion of the serum amyloid P component gene. *Nature Medicine* **3**, 855-859
75. Duong, T., Pommier, E. C., and Scheibel, A. B. (1989) Immunodetection of the amyloid P component in Alzheimer's disease. *Acta Neuropathol* **78**, 429-437
76. Flo, T. H., Smith, K. D., Sato, S., Rodriguez, D. J., Holmes, M. A., Strong, R. K., Akira, S., and Aderem, A. (2004) Lipocalin 2 mediates an innate immune response to bacterial infection by sequestrating iron. *Nature* **432**, 917-921

77. Sousa, M. M., do Amaral, J. B., Guimaraes, A., and Saraiva, M. J. (2005) Up-regulation of the extracellular matrix remodeling genes, biglycan, neutrophil gelatinase-associated lipocalin, and matrix metalloproteinase-9 in familial amyloid polyneuropathy. *Faseb J* **19**, 124-126
78. Naude, P. J., Dekker, A. D., Coppus, A. M., Vermeiren, Y., Eisel, U. L., van Duijn, C. M., Van Dam, D., and De Deyn, P. P. (2015) Serum NGAL is Associated with Distinct Plasma Amyloid-beta Peptides According to the Clinical Diagnosis of Dementia in Down Syndrome. *J Alzheimers Dis* **45**, 733-743
79. Hoeksema, M., van Eijk, M., Haagsman, H. P., and Hartshorn, K. L. (2016) Histones as mediators of host defense, inflammation and thrombosis. *Future Microbiol* **11**, 441-453
80. Narayan, P. J., Lill, C., Faull, R., Curtis, M. A., and Dragunow, M. (2015) Increased acetyl and total histone levels in post-mortem Alzheimer's disease brain. *Neurobiol Dis* **74**, 281-294
81. Biedzka-Sarek, M., Metso, J., Kateifides, A., Meri, T., Jokiranta, T. S., Muszynski, A., Radziejewska-Lebrecht, J., Zannis, V., Skurnik, M., and Jauhiainen, M. (2011) Apolipoprotein A-I exerts bactericidal activity against Yersinia enterocolitica serotype O:3. *J Biol Chem* **286**, 38211-38219
82. Gupta, H., Dai, L., Datta, G., Garber, D. W., Grenett, H., Li, Y., Mishra, V., Palgunachari, M. N., Handattu, S., Gianturco, S. H., Bradley, W. A., Anantharamaiah, G. M., and White, C. R. (2005) Inhibition of lipopolysaccharide-induced inflammatory responses by an apolipoprotein AI mimetic peptide. *Circ Res* **97**, 236-243
83. Tada, N., Sakamoto, T., Kagami, A., Mochizuki, K., and Kurosaka, K. (1993) Antimicrobial activity of lipoprotein particles containing apolipoprotein Al. *Mol Cell Biochem* **119**, 171-178
84. Obici, L., Franceschini, G., Calabresi, L., Giorgetti, S., Stoppini, M., Merlini, G., and Bellotti, V. (2006) Structure, function and amyloidogenic propensity of apolipoprotein A-I. *Amyloid* **13**, 191-205
85. Westermark, P., Mucchiano, G., Marthin, T., Johnson, K. H., and Sletten, K. (1995) Apolipoprotein A1-derived amyloid in human aortic atherosclerotic plaques. *Am J Pathol* **147**, 1186-1192
86. Nichols, W. C., Dwulet, F. E., Liepnieks, J., and Benson, M. D. (1988) Variant Apolipoprotein-a-I as a Major Constituent of a Human Hereditary Amyloid. *Biochem Bioph Res Co* **156**, 762-768
87. Merched, A., Xia, Y., Visvikis, S., Serot, J. M., and Siest, G. (2000) Decreased high-density lipoprotein cholesterol and serum apolipoprotein AI concentrations are highly correlated with the severity of Alzheimer's disease. *Neurobiol Aging* **21**, 27-30
88. Lewis, T. L., Cao, D., Lu, H., Mans, R. A., Su, Y. R., Jungbauer, L., Linton, M. F., Fazio, S., LaDu, M. J., and Li, L. (2010) Overexpression of human apolipoprotein A-I preserves cognitive function and attenuates neuroinflammation and cerebral amyloid angiopathy in a mouse model of Alzheimer disease. *J Biol Chem* **285**, 36958-36968
89. Endres, K. (2021) Apolipoprotein A1, the neglected relative of Apolipoprotein E and its potential role in Alzheimer's disease. *Neural Regen Res* **16**, 2141-2148

**Figure S1**

**Protein aggregation in AWFs challenged by LPS, related to Figure 2.** A) LPS was mixed with AWFs followed by centrifugation and analysis of LPS levels in the supernatant (LPS s) and pellet (LPS p). Non-centrifuged samples were used for control (LPS). A LAL assay demonstrated a significant decrease in LPS concentration in the supernatants of LPS-treated AWFs after centrifugation (LPS s, left panel), while an increase was observed in the pellets (LPS p, right panel), relative the non-centrifuged samples (LPS). Statistical analysis was performed using one-way ANOVA with Dunnett's multiple comparison tests based on data from four independent experiments (n=3). *= $P \leq 0.05$, **= $P \leq 0.01$ and *** = $P \leq 0.001$. B) In the ThT aggregation assay, a significant increase in ThT fluorescence was observed in three out of four AWFs after the addition of 100 µg/ml of LPS from *Pseudomonas aeruginosa* (Pa). No increase in ThT signal was seen in the presence of lipid A, and in both cases, no significant effects on aggregation were observed in the CP samples. Statistical analysis was performed using one-way ANOVA with Dunnett's multiple comparison tests from four independent experiments (n=4). *= $P \leq 0.05$ and ** = $P \leq 0.01$. C) TEM-negative stain analysis revealed the presence of amorphous aggregates in all AWFs exposed to LPS (100 µg/ml). The images represent an representative example from three independent experiments. The scale bar corresponds to 5 µm.

**Figure S2**

**Image analyses of Amytracker 680 signal in AWFs, related to Figure 2.** A) Statistical analysis was performed using one-way ANOVA with Dunnett's multiple comparison tests from three independent experiments (n=3). * = $P \leq 0.05$, ** = $P \leq 0.01$, **** = $P \leq 0.0001$, ns = not significant. B) Fluorescence microscopy analysis using Amytracker 680 stain. No protein aggregation in buffer or LPS alone is detected. The scale bar is 5 µm. The images represent an example from three independent experiments. The scale bar corresponds to 5 µm.

**Figure S3**

**Fluorescence microscopy, related to Figure 2.** Fluorescence microscopy revealed the presence of protein aggregates in AWFs exposed to FITC-LPS (100 µg/ml) (green) and stained with Amytracker 680 stain (red). The images represent a representative example from three independent experiments. The scale bar corresponds to 5 µm.

**Figure S4**

**A clustered* heatmap of the proteomic content of the pellet and supernatant, related to Figure 4.** The color represents the relative intensity, i.e., *($I_p/I_{total}$),* where $I_p$ is the protein intensity in the pellet or supernatant respectively, and $I_{total}$ is the total intensity in both the pellet and supernatant for the given protein. The map was clustered on both samples and proteins. Proteins not found in 2 samples were discarded (evaluated for the pellet and supernatant individually).

*Clustering was performed using the average Euclidian distance on the relative protein abundances.

**Figure S5**

**NF-κB activation assay, related to Figure 6.** THP-1 cells pre-treated with LPS followed by washing (denoted LPS w), and subsequently treated with the different AWFs (AWF) showed no significant reduction of NF-κB activation relative pre-treatment with LPS alone. Addition of lepirudin plasma (LP) did not result in a reduction of LPS-induced NF-κB activation. Tris buffer (Buffer) was used as negative control. The positive control TCP-25 inhibited LPS induced NF-κB activation. A setup with continuous LPS stimulation (LPS) is also shown for comparison. MTT viability assay showed no toxic effect of LPS, AWFs or LP on THP-1 cells. Statistical analysis was performed using one-way ANOVA with Dunnett's multiple comparison tests from four independent experiments (n=4). ** = $P \leq 0.01$,*** = $P \leq 0.001$, ns = not significant. B) A phagocytosis assay, conducted using the macrophage cell line RAW 264.7, revealed a significant increase in phagocytosis of protein aggregates in AWFs stained with Amytracker 680. Statistical analysis was performed using one-way ANOVA with Dunnett's multiple comparison tests from four independent experiments (n=4). ** = $P \leq 0.01$,*** = $P \leq 0.001$, ns = not significant. C) Fluorescence microscopy imaging of protein aggregates was conducted using AWF1 pre-treated with LPS (FITC, green) and stained with Amytracker 680 (red). RAW 264.7 cells were utilized for the experiment, and DAPI staining was applied to visualize the cell nuclei (blue). The images represent an representative example from three independent experiments. The scale bar corresponds to 5 μm.

**Figure S6**

**Inhibition of LPS stimulation, related to Figure 6.** A) THP1 cells were stimulated with LPS alone, or LPS aggregated by the different AWFs and TNF-α (left panel) and IL-1β levels (right

panel) were analyzed. Addition of AWFs to LPS yielded a significant reduction of the two cytokines. TAK-242 (TAK) addition to this combination did not yield a statistically significant further reduction. TAK-242 and TCP-25 alone served as positive controls. Statistical analysis was performed using one-way ANOVA with Dunnett's multiple comparison tests from three independent experiments (n=3). *** = P≤ 0.001, ns = not significant. B) The schematic figure illustrates AWF-mediated LPS-scavenging and sequestration in inhibiting downstream effects on TLR-4 dependent cytokines, while also depicting the inhibitory action of TAK-242 and TCP-25, both acting downstream of the scavenging mechanism.

Figure S1

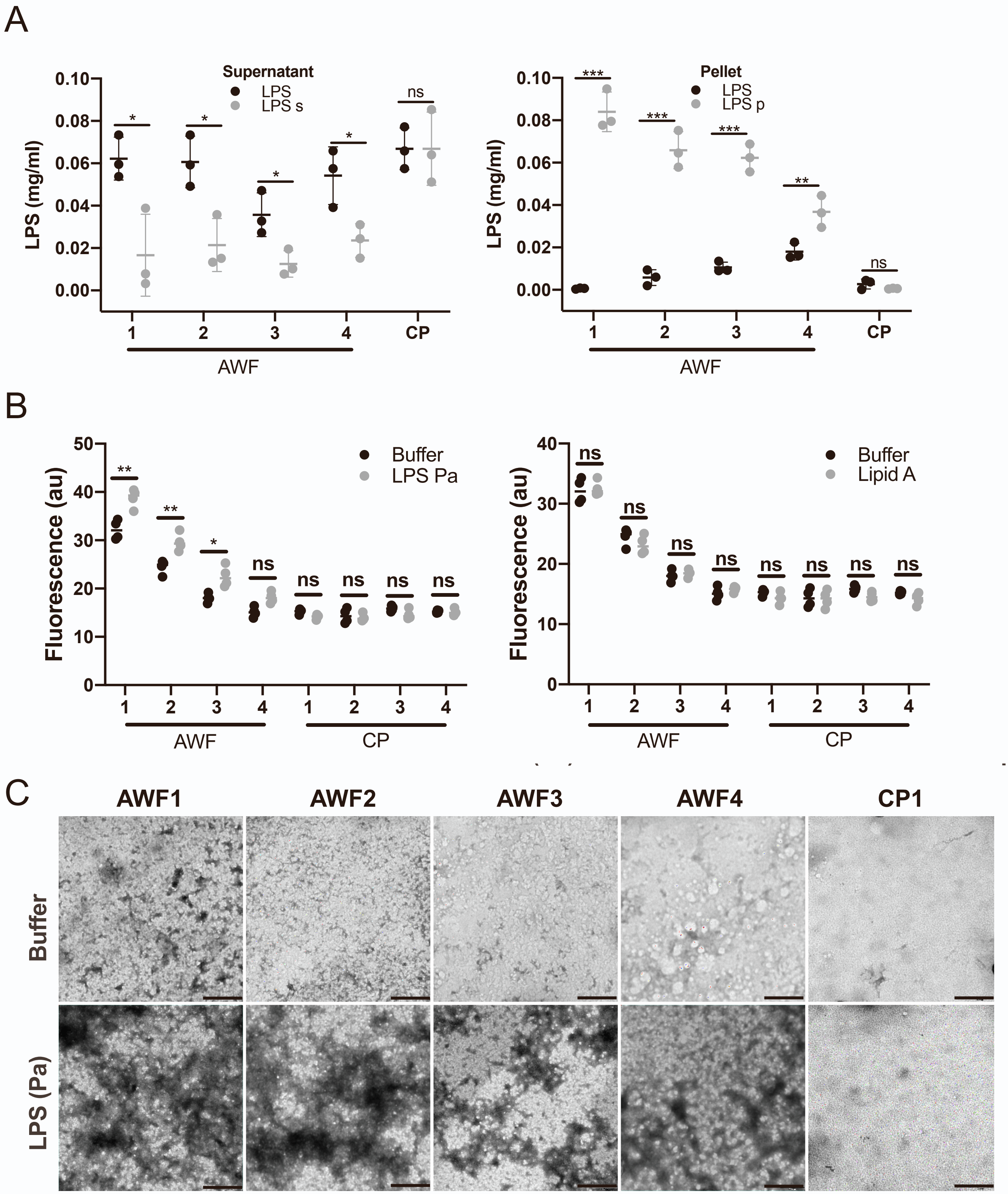
A
Supernatant
LPS
LPS s
LPS (mg/ml)
0.10
0.08
0.06
0.04
0.02
0.00
*
ns
1
2
3
4
CP
AWF
Pellet
LPS
LPS p
***
**
ns
B
Buffer
LPS Pa
Fluorescence (au)
50
40
30
20
10
0
AWF
CP
Buffer
Lipid A
C
AWF1
AWF2
AWF3
AWF4
CP1
Buffer
LPS (Pa)

Figure S2

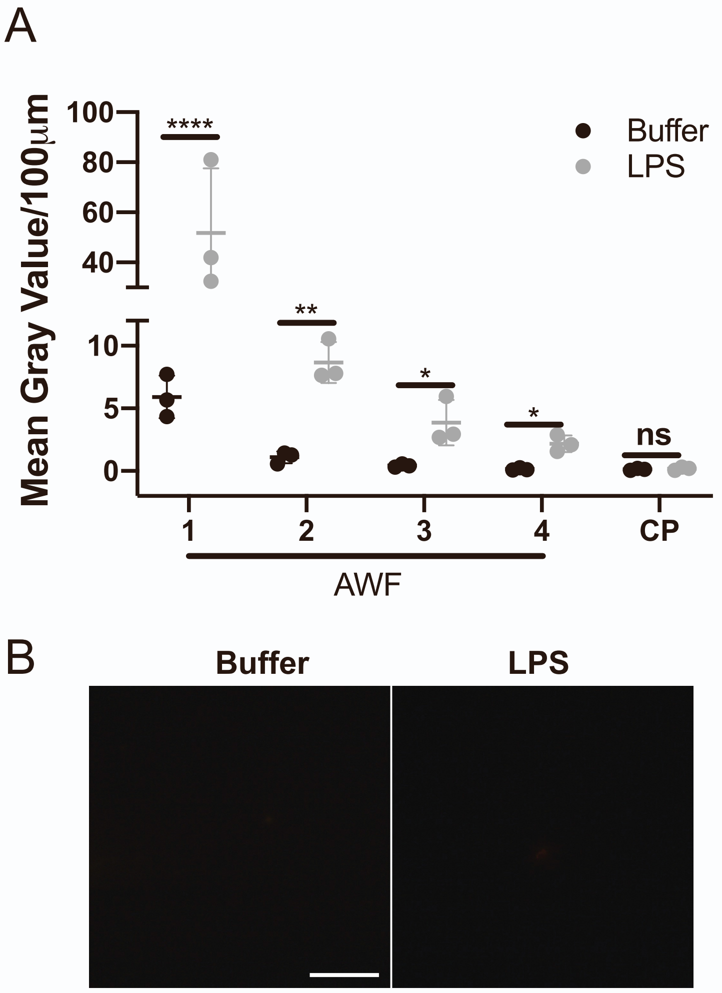
A
Mean Gray Value/100μm
100
80
60
40
10
5
0
****
**
*
*
ns
Buffer
LPS
1
2
3
4
CP
AWF
B
Buffer
LPS

Figure S3

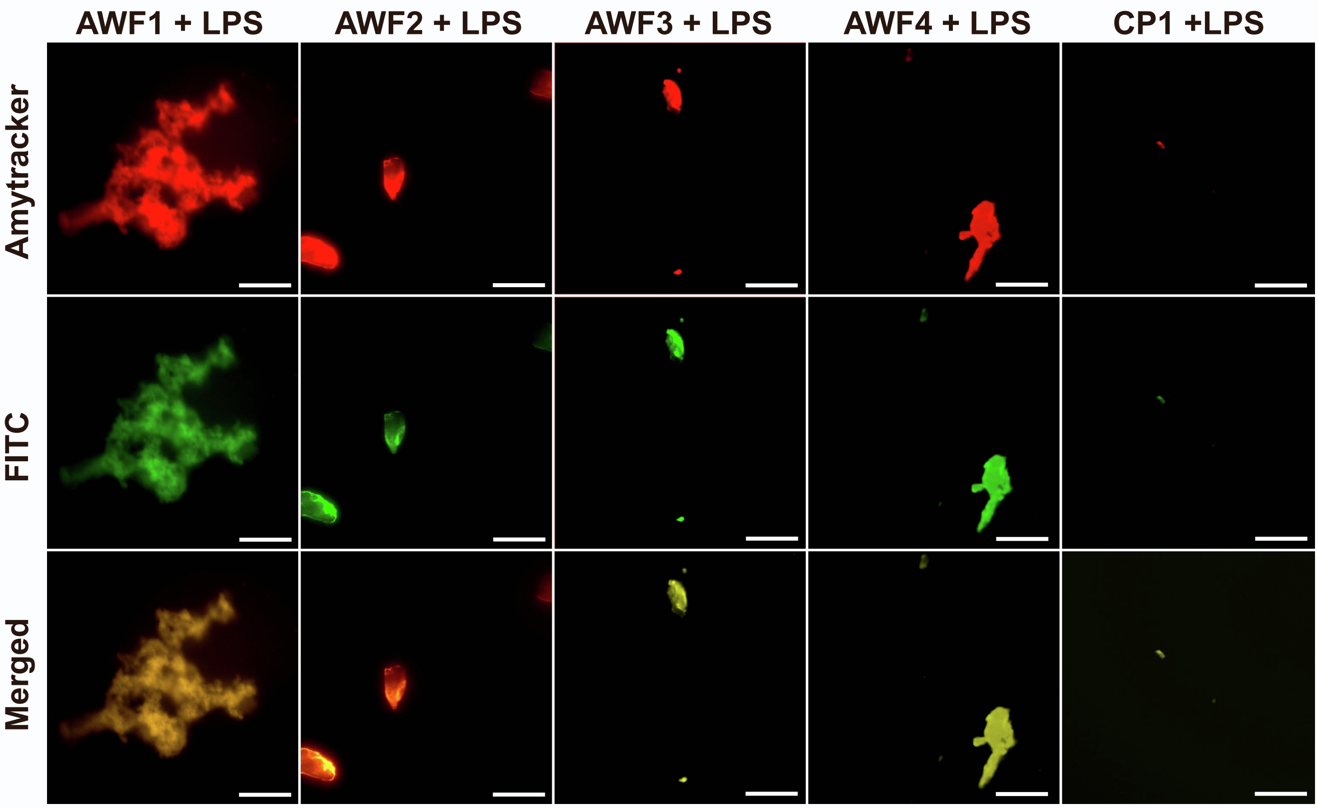
AWF1 + LPS
AWF2 + LPS
AWF3 + LPS
AWF4 + LPS
CP1 +LPS
Amytracker
FITC
Merged

Figure S4

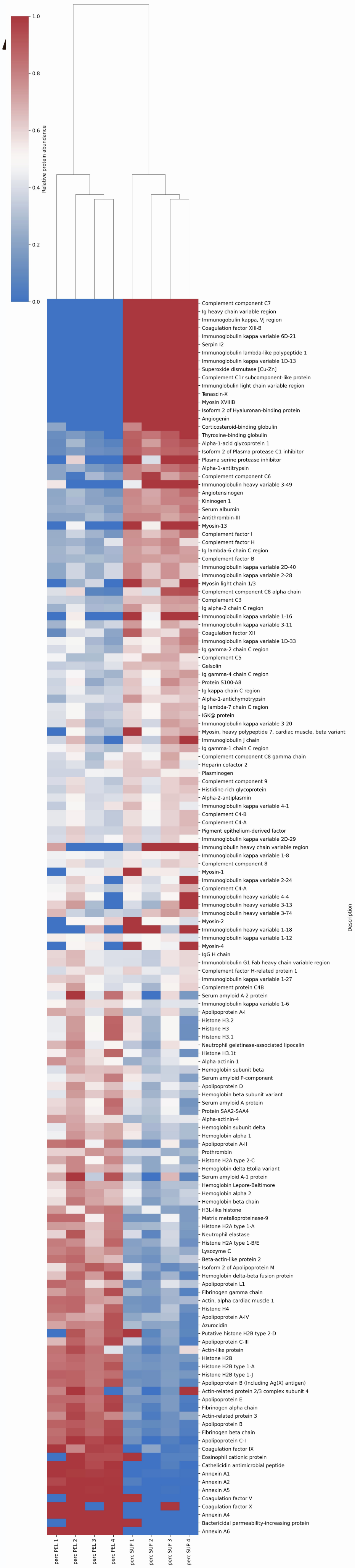

1.0
0.8
0.6
0.4
0.2
0.0
Relative protein abundance
Complement component C7
Ig heavy chain variable region
Immunogobulin kappa, VJ region
Coagulation factor XIII-B
Immunoglobulin kappa variable 6D-21
Serpin I2
Immunoglobulin lambda-like polypeptide 1
Immunoglobulin kappa variable 1D-13
Superoxide dismutase [Cu-Zn]
Complement C1r subcomponent-like protein
Immunglobulin light chain variable region
Tenascin-X
Myosin XVIIIB
Isoform 2 of Hyaluronan-binding protein
Angiogenin
Corticosteroid-binding globulin
Thyroxine-binding globulin
Alpha-1-acid glycoprotein 1
Isoform 2 of Plasma protease C1 inhibitor
Plasma serine protease inhibitor
Alpha-1-antitrypsin
Complement component C6
Immunoglobulin heavy variable 3-49
Angiotensinogen
Kininogen 1
Serum albumin
Antithrombin-III
Myosin-13
Complement factor I
Complement factor H
Ig lambda-6 chain C region
Complement factor B
Immunoglobulin kappa variable 2D-40
Immunoglobulin kappa variable 2-28
Myosin light chain 1/3
Complement component C8 alpha chain
Complement C3
Ig alpha-2 chain C region
Immunoglobulin kappa variable 1-16
Immunoglobulin kappa variable 3-11
Coagulation factor XII
Immunoglobulin kappa variable 1D-33
Ig gamma-2 chain C region
Complement C5
Gelsolin
Ig gamma-4 chain C region
Protein S100-A8
Ig kappa chain C region
Alpha-1-antichymotrypsin
Ig lambda-7 chain C region
IGK@ protein
Immunoglobulin kappa variable 3-20
Myosin, heavy polypeptide 7, cardiac muscle, beta variant
Immunoglobulin J chain
Ig gamma-1 chain C region
Complement component C8 gamma chain
Heparin cofactor 2
Plasminogen
Complement component 9
Histidine-rich glycoprotein
Alpha-2-antiplasmin
Immunoglobulin kappa variable 4-1
Complement C4-B
Complement C4-A
Pigment epithelium-derived factor
Immunoglobulin kappa variable 2D-29
Immunglobulin heavy chain variable region
Immunoglobulin kappa variable 1-8
Complement component 8
Myosin-1
Immunoglobulin kappa variable 2-24
Complement C4-A
Immunoglobulin heavy variable 4-4
Immunoglobulin heavy variable 3-13
Immunoglobulin heavy variable 3-74
Myosin-2
Immunoglobulin heavy variable 1-18
Immunoglobulin kappa variable 1-12
Myosin-4
IgG H chain
Immunoblobulin G1 Fab heavy chain variable region
Complement factor H-related protein 1
Immunoglobulin kappa variable 1-27
Complement protein C4B
Serum amyloid A-2 protein
Immunoglobulin kappa variable 1-6
Apolipoprotein A-I
Histone H3.2
Histone H3
Histone H3.1
Neutrophil gelatinase-associated lipocalin
Histone H3.1t
Alpha-actinin-1
Hemoglobin subunit beta
Serum amyloid P-component
Apolipoprotein D
Hemoglobin beta subunit variant
Serum amyloid A protein
Protein SAA2-SAA4
Alpha-actinin-4
Hemoglobin subunit delta
Hemoglobin alpha 1
Apolipoprotein A-II
Prothrombin
Histone H2A type 2-C
Hemoglobin delta Etolia variant
Serum amyloid A-1 protein
Hemoglobin Lepore-Baltimore
Hemoglobin alpha 2
Hemoglobin beta chain
H3L-like histone
Matrix metalloproteinase-9
Histone H2A type 1-A
Neutrophil elastase
Histone H2A type 1-B/E
Lysozyme C
Beta-actin-like protein 2
Isoform 2 of Apolipoprotein M
Hemoglobin delta-beta fusion protein
Apolipoprotein L1
Fibrinogen gamma chain
Actin, alpha cardiac muscle 1
Histone H4
Apolipoprotein A-IV
Azurocidin
Putative histone H2B type 2-D
Apolipoprotein C-III
Actin-like protein
Histone H2B
Histone H2B type 1-A
Histone H2B type 1-J
Apolipoprotein B (Including Ag(X) antigen)
Actin-related protein 2/3 complex subunit 4
Apolipoprotein E
Fibrinogen alpha chain
Actin-related protein 3
Apolipoprotein B
Fibrinogen beta chain
Apolipoprotein C-I
Coagulation factor IX
Eosinophil cationic protein
Cathelicidin antimicrobial peptide
Annexin A1
Annexin A2
Annexin A5
Coagulation factor V
Coagulation factor X
Annexin A4
Bactericidal permeability-increasing protein
Annexin A6
Description
perc PEL 1
perc PEL 2
perc PEL 3
perc PEL 4
perc SUP 1
perc SUP 2
perc SUP 3
perc SUP 4

Figure S5

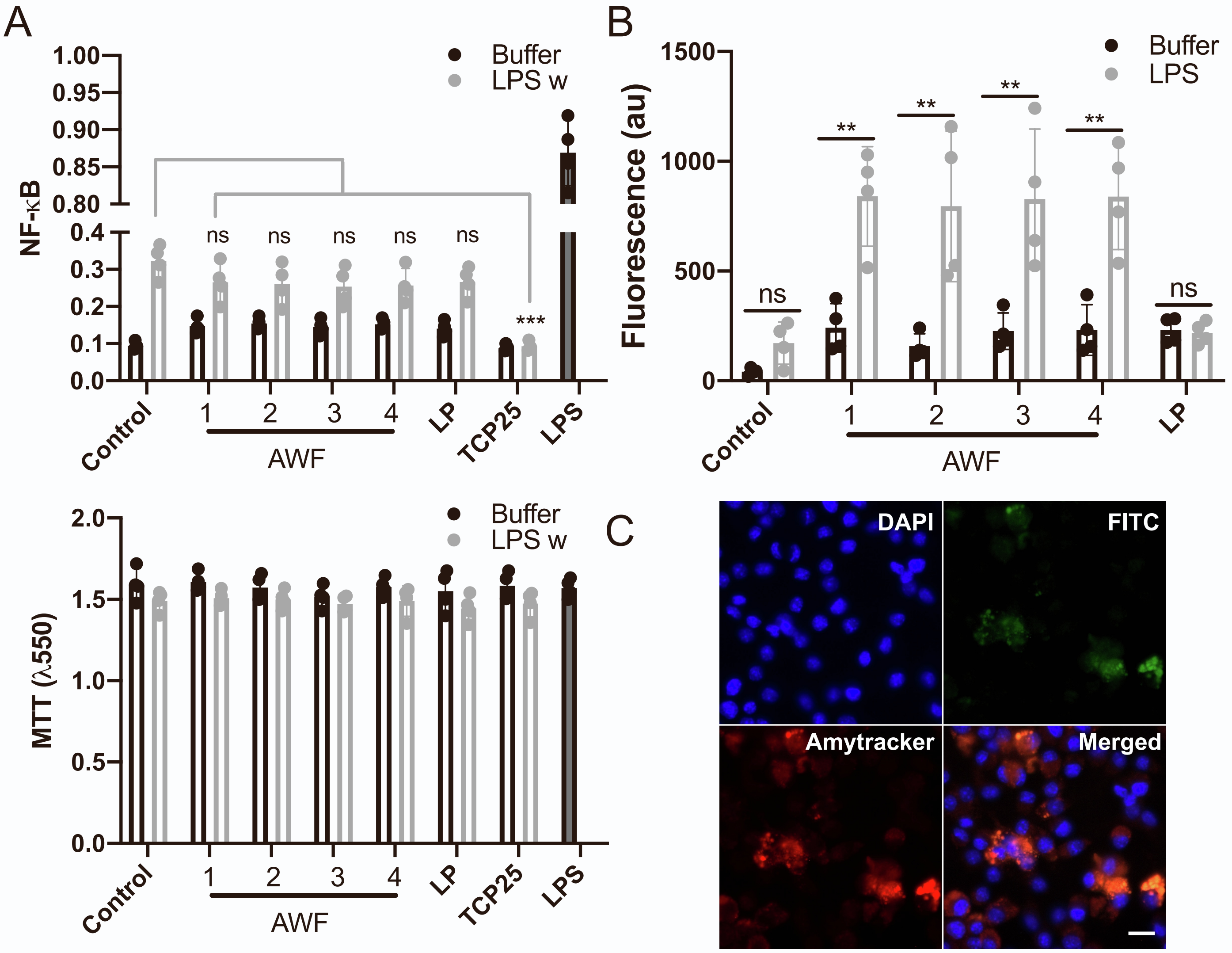
A
NF-κB
1.00
0.95
0.90
0.85
0.80
0.4
0.3
0.2
0.1
0.0
Buffer
LPS w
ns
ns
ns
ns
ns
***
Control
1
2
3
4
AWF
LP
TCP25
LPS
B
Fluorescence (au)
1500
1000
500
0
Buffer
LPS
ns
**
**
**
**
ns
Control
1
2
3
4
AWF
LP
MTT (λ550)
2.0
1.5
1.0
0.5
0.0
Buffer
LPS w
Control
1
2
3
4
AWF
LP
TCP25
LPS
C
DAPI
FITC
Amytracker
Merged

Figure S6

A

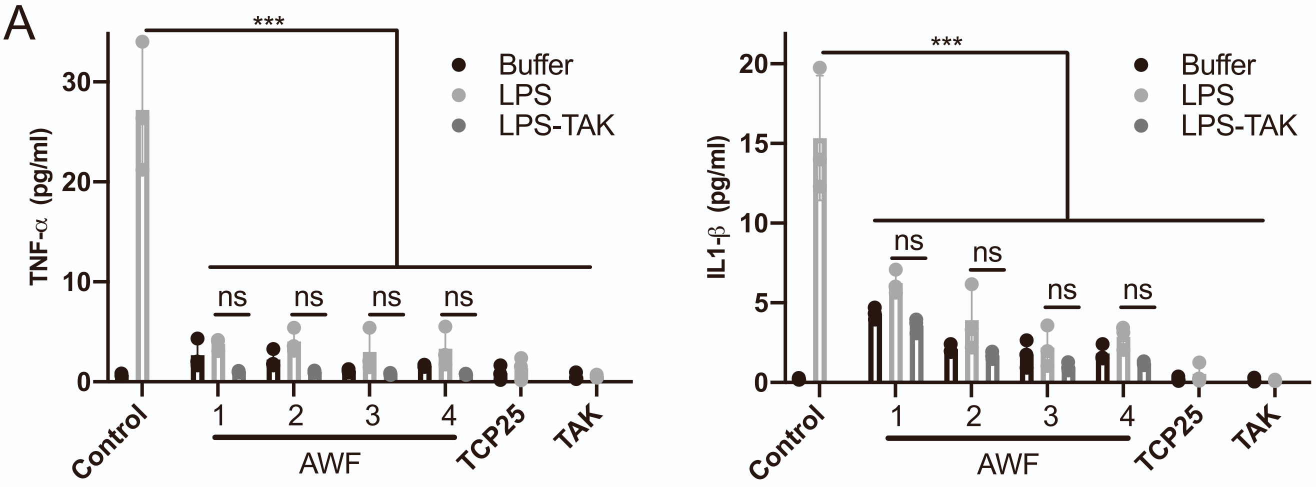

***
Buffer
LPS
LPS-TAK
TNF-α (pg/ml)
30
20
10
0
ns
ns
ns
ns
Control
1
2
3
4
AWF
TCP25
TAK
***
Buffer
LPS
LPS-TAK
IL1-β (pg/ml)
20
15
10
5
0
ns
ns
ns
ns
Control
1
2
3
4
AWF
TCP25
TAK


B

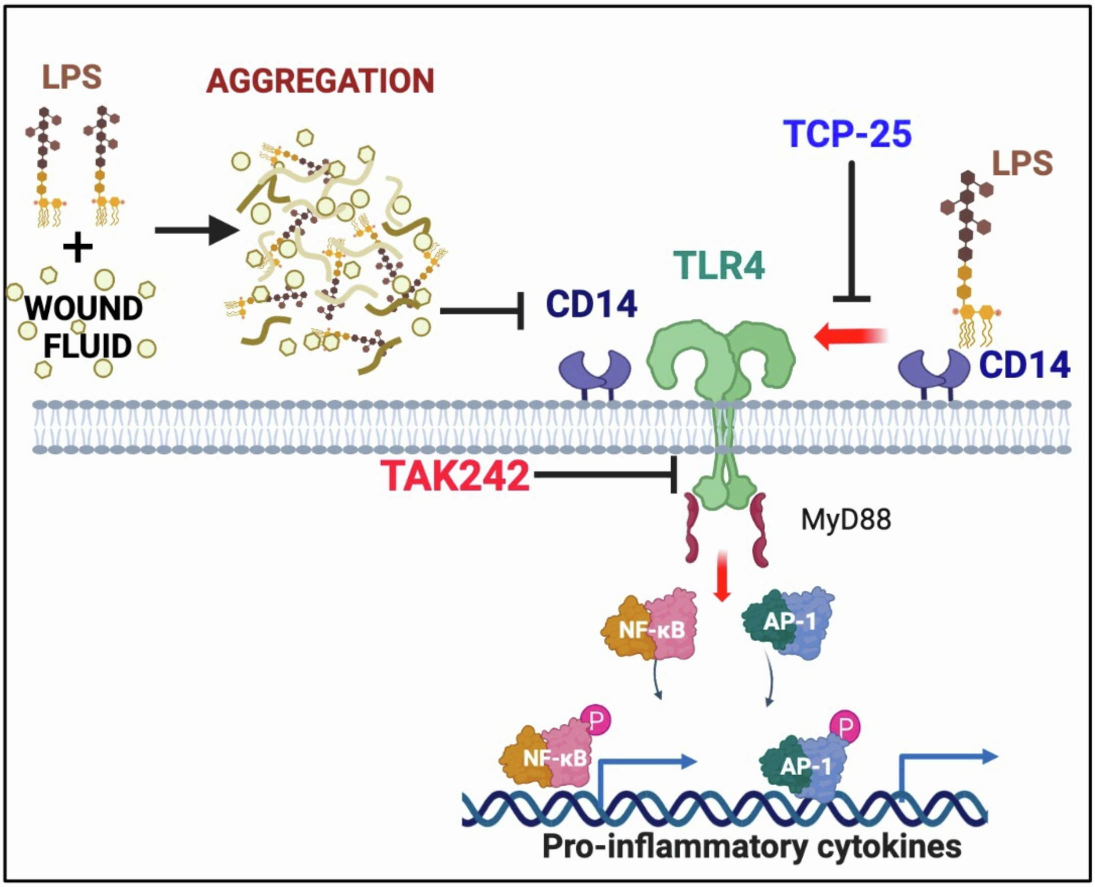

LPS
AGGREGATION
+
WOUND
FLUID
TCP-25
LPS
TLR4
CD14
CD14
TAK242
MyD88
NF-κB
AP-1
NF-κB
AP-1
P
P
Pro-inflammatory cytokines
